# Targeted delivery of *BACE1* siRNA for synergistic treatment of Alzheimer's disease

**DOI:** 10.1186/s40035-025-00503-7

**Published:** 2025-08-14

**Authors:** Zhaohan Li, Jun Yang, Jianan Li, Shuxuan Zhao, Shaoping Jiang, Weimin Liu, Xinjian Li, Simeng Zhang, Haiyan Du, Junjun Ni, Yuanyu Huang, Hong Qing, Shaobo Ruan

**Affiliations:** 1 School of Life Science, Beijing Institute of Technology, Beijing, 100081 China; 2 School of Interdisciplinary Science, Beijing Institute of Technology, Beijing, 100081 China; 3 Department of Cell Biology, Aging Science, and Pharmacology, Division of Oral BiologicalSciences, Faculty of Dental Science, Kyushu University, 3-1-1 Maidashi, Higashi-Ku, Fukuoka, 812-8582 Japan; 4 Shenzhen MSU-BIT University, No.1, International University Park Road, Dayun New Town, Longgang District, Shenzhen, China

**Keywords:** Microglia, Alzheimer's disease, Nanomedicine, siRNA, Blood–brain barrier, BACE1, Aβ, Neuroinflammation

## Abstract

**Background:**

The deposition of toxic aggregated amyloid-β (Aβ), resulting from continuous cleavage of amyloid precursor protein (APP) by β-site APP cleaving enzyme 1 (BACE1) and γ-secretase, is a key pathogenic event in Alzheimer's disease (AD). Small interfering RNAs (siRNA) have shown great potential for disease treatment by specifically silencing target genes. However, the poor brain delivery efficiency of siRNAs limits their therapeutic efficacy against AD.

**Methods:**

We designed a simplified and effective *BACE1* siRNA (siBACE1) delivery system, namely, dendritic polyamidoamine modified with the neurotropic virus-derived peptide RVG29 and polyethylene glycol (PPR@siBACE1).

**Results:**

PPR@siBACE1 crossed the blood–brain barrier efficiently and entered brain parenchyma in large amount, with subsequent neurotropism and potential microglia-targeting ability. Both in vitro and in vivo studies validated the effective brain delivery of siBACE1 and strong *BACE1* silencing efficiency. Treatment of AD mice with PPR@siBACE1 inhibited the production of Aβ, potentiated Aβ phagocytosis by microglia, improved the memory deficits and reduced neuroinflammatory response in AD mice.

**Conclusions:**

This study provides a reliable delivery platform for gene therapies for AD.

**Supplementary Information:**

The online version contains supplementary material available at 10.1186/s40035-025-00503-7.

## Background

Alzheimer's disease (AD) is the most common neurodegenerative disease accounting for approximately 60%–70% of all dementias globally. AD is closely related to aging and is more prevalent in individuals aged 65 and above. The clinical symptoms of AD include impaired memory and cognitive function, along with aberrant behavior and social dysfunction. According to the World Health Organization, approximately 50 million people globally were affected by AD in 2019, and this figure is projected to rise to 152 million by 2050 [1]. The main treatment for AD involves pharmacological interventions including cholinesterase inhibitors, *N*-methyl-_*D*_-aspartate receptor antagonists and a combination preparation. However, these drugs can only alleviate symptoms and provide temporary relief for patients by improving their cognitive ability during medications [2]. Furthermore, they are unable to prevent the progression of AD, with no reductions of progression of disability or the rate of hospitalization following treatment course [3]. Thus, disease-modifying therapies targeting the pathogenic factors of AD are highly needed [4–6].

To date, the pathogenesis of AD remains unclarified and is still under debate. Several pathological features have been identified, including abnormal deposition of amyloid-β (Aβ) plaques, neurofibrillary tangles, neuroinflammatory response, decreased acetylcholine levels and oxidative stress [7]. All these features have been confirmed to be involved in the onset and progression of AD. Among these, deposition of Aβ plaques plays a dominant role in driving the pathogenesis of AD and triggering a cascade of pathological progression [8]. The amyloid cascade hypothesis posits that in the context of AD, amyloid precursor protein (APP) is predominantly hydrolyzed by β-site APP cleavage enzyme 1 (BACE1) and γ-secretase, leading to the production of soluble Aβ monomers, which subsequently form toxic oligomers or fibrils. The Aβ fibrils accumulate within the brain, eventually forming Aβ plaques, causing synaptic damage and neuronal apoptosis. They also promote excessive phosphorylation of tau protein, ultimately leading to the formation of neurofibrillary tangles and the onset of AD [9]. Recently, the approval of three monoclonal antibodies that target Aβ-related plaques, fibrils or oligomers has guided increasing attention on Aβ-related pathogenesis as a therapeutic target [10]. Given that elevated neuronal BACE1 activity is closely relevant with Aβ generation in the brains of AD patients, inhibition of BACE1 is recognized as a promising alternative strategy, which can reduce the deposition of Aβ directly [11].

Currently, various small-molecule inhibitors of BACE1 have been developed, of which 5 are undergoing clinical investigation. Despite a reduction of Aβ load, AD patients receiving these medications failed to show cognitive benefits, probably because BACE1 inhibition does not influence pre-existing Aβ. Therefore, inhibiting BACE1 while simultaneously clearing the already existing Aβ is extremely important to modify the progression of AD [12, 13]. Recent research has identified genetically distinct subtypes of microglia, termed homeostatic microglia and disease-associated microglia (DAM), which react to pathological changes in the brain microenvironment of AD patients [14–16]. Specifically, stage 1 DAM (DAM-1), represents a more robust and phagocytosis-capable subtype, whereas stage 2 DAM (DAM-2) represents a phagocytosis-dysfunctional and inflammation-related subtype linked to AD pathology [17]. Importantly, a recent study revealed that genetic knockout of BACE1 in microglia not only preserves the DAM-1 state, but also enhances the Aβ-clearance capability of microglia [18]. Therefore, inhibiting BACE1 activity in both neurons and microglia may make it possible to simultaneously prevent Aβ generation and clear pre-existing Aβ [19, 20].

RNA interference (RNAi) is a powerful gene silencing technique that can specifically knock down the expression of target genes via complementary nucleotide sequences. Small interference RNA (siRNA), microRNA, and inhibitory antisense oligonucleotides are representative RNA molecules for gene silencing [21–23]. Notably, siRNA has been most extensively studied and exhibits several advantages over conventional small molecules, such as high specificity, high druggability, durable inhibitory effects, reduced dosage requirements, and manageable side effects, making it a potent therapeutic tool for inhibiting BACE1 expression [24, 25]. However, the use of naked siRNA for AD treatment faces several challenges if administered systemically, including readily degradation by nucleases, short blood circulation time, potential immunogenicity [26–29], and the lack of ability to cross biological barriers, particularly blood–brain barrier (BBB) [30]. Fortunately, the advancement of nanocarrier-based brain targeting delivery system offers the opportunity to troubleshoot these issues. Particularly, these nanocarriers with positive charges can efficiently encapsulate negatively charged siRNA by electrostatic absorption, protecting siRNA from degradation and leakage [31]. Moreover, through modification with brain-targeting ligands, nanocarriers can specifically recognize receptors or transporters expressed on both BBB and neurons to achieve efficient and precise brain delivery of siRNA [32].

**Fig. 1:**
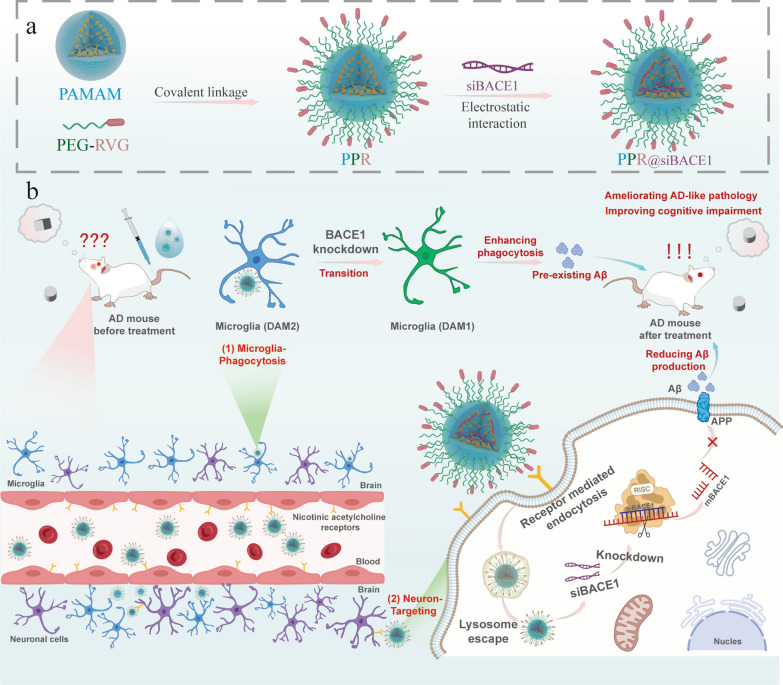
Preparation, delivery and pharmacological action of PPR@siBACE1 for AD treatment. **a** Illustration of the composition, assembly, and siRNA encapsulation of PPR@siBACE1. **b** Illustration of targeted delivery of PPR@siBACE1 to neurons (in a nAchR-dependent manner) and microglia (primarily in a nAchR-independent manner), as well as the synergistic pharmacological action by genetic knockdown of BACE1 in both neurons and microglia

In this study, we proposed a brain-targeting siRNA delivery system by using polyamidoamine (PAMAM), a cationic dendrimer with abundant amino groups, as a carrier. The PAMAM was modified with polyethylene glycol (PEG)-covalently-linked rabies virus protein 29 (RVG29) peptide, and loaded with *BACE1* siRNA (siBACE1) [33, 34]. RVG29 polypeptide is a targeted ligand consisting of 29 polypeptides derived from the rabies virus glycoprotein, which can recognize nicotinic acetylcholine receptors (nAchRs) on the BBB [33–36]. The delivery system PPR@siBACE1 can recognize nAchRs expressed on the BBB and effectively cross BBB through nAchR-mediated transcytosis [37]. After entering brain parenchyma, PPR@siBACE1 can further recognize nAchR on neurons, enabling targeted delivery to neurons. The PPR@siBACE1 also has the chance to be phagocytosed by microglia, the brain-resident macrophages with strong phagocytic capability. Our key design elements include: (1) BBB penetration with optimized nanoparticle physicochemical properties, (2) cell-specific cargo delivery via receptor-mediated targeting, and (3) therapeutic synergy through simultaneous intervention in Aβ production and clearance. The design rationale is based on the pathological mechanisms: neuronal BACE1 promotes Aβ production, while microglial BACE1 affects phagocytic function. Through dual regulation, this approach achieves synergistic therapeutic effects: suppressing neuronal BACE1 to reduce Aβ generation and modulating microglial BACE1 to enhance Aβ clearance (Fig. 1).

## Materials and methods

### Materials

Two-month-old female C57BL/6J wild-type (WT) mice, provided by SBI Biotechnology Co., Ltd (Beijing, China), were used for studying biodistribution. Eight-month-old male FAD^4T^ mice, provided by Jicui Pharmaceutical Co., Ltd. (Nanjing, China), were used for drug treatment. All mice were sent to the animal facility of Beijing Institute of Technology at least 2 weeks prior to experiments. Mice were raised in accordance with the Guidelines for the Management and Use of Laboratory Animals. Mice were housed 4–6 in each cage at 25°C, under a 12-h circadian rhythm with free access to food and water. All experimental procedures were conducted during 10:00 and 19:00. All experimenters held the Beijing Laboratory Animal Practitioner Certificate. The sequences of siRNAs (Biosyntech, Suzhou, China) are as follows: sense strand of the siRNA capable of specifically silencing *BACE1*, 5'-UGGACUGCAAGGAGUACAA-3'; antisense strand, 5'-UUGUACUCCUUGCAGUCCAUU-3'; sense strand of the negative control siRNA, 5'-UUCUCCGAACGUGUCACGUTT-3', antisense strand 5'-ACGUGACACGUUCGGAGAATT-3'. The fluorescent siRNA used in the experiment was the negative control siRNA labeled with Cy5. Antibodies used in this study included: primary antibodies for Aβ (803014, Biolegends, San Diego, CA), glial fibrillary acidic protein (GFAP) (AB53554, Abcam, Cambridge, UK), Iba1 (019-19741, Wako, Osaka, Japan), Nissl (N21482, Thermo Fisher Scientific, Waltham, MA), myelin basic protein (MBP) (MCA409S, BioRad, Hercules, CA), BACE1 (AB183612, Abcam), β-actin (AB8226, Abcam). Secondary antibodies were purchased from Abcam (AB6721, AB6741, AB205719, AB150135, AB150074, AB150105). All qPCR primer sequences are listed in Table S1.

### Preparation of siRNA nanomedicines

siRNA dry powder was centrifuged at 1000 rpm for 5 min at 4°C, then an appropriate amount of DEPC water was added to prepare the siRNA solution. PAMAM-PEG (PP) or PAMAM-PEG-RVG29 (PPR) (preparation method provided in Supplementary Methods) dry powder was dissolved in DEPC water, then the siRNA solution was added dropwise in a specific ratio, and mixed immediately for 30 s. The resulting sample was left at room temperature for 20 min. The nanomedicine was prepared based on the mass ratio of PAMAM to siRNA (20:1, 15:1, 10:1, 5:1, and 1:1). The final products were the targeted nanomedicine PPR@siRNA and the non-targeted nanomedicine PP@siRNA, stored at 4°C.

### Characterization of particle size and zeta potential

The targeting nanomaterial PPR, non-targeting nanomaterial PP, as well as the targeting nanomedicine PPR@siRNA and non-targeting nanomedicine PP@siRNA were analyzed for particle size and zeta potential by dynamic laser scattering (DLS) using a zetasizer (Malvern Instruments, Worcestershire, UK) at 25°C. Measurements were repeated for three times.

### Gel retardation electrophoresis

To assess the effectiveness of the nano-drugs prepared above in siRNA loading, agarose electrophoresis was performed. Initially, PPR with PAMAM-to-siRNA mass ratios of 20:1, 15:1, 10:1, 5:1, and 1:1 were prepared. Subsequently, 12 μL of the nano-drug solution was mixed with 3 μL of loading buffer (Merck, Darmstadt, Germany) and loaded onto a 2% agarose gel. The sample then underwent 80 V constant voltage electrophoresis in 1×Tris-Acetate-EDTA gel electrophoresis (TAE) (50×: 242 g/L Tris-base, 57.1 mL/L glacial acetic acid, 37.2 g/L EDTA-Na₂, pH 8.3) for 10 min, and observed with a gel imager (iBright CL1500, Thermo Fisher Scientific).

### Morphological characterization by transmission electron microscopy (TEM)

Briefly, 10 μL of freshly prepared PPR@siRNA and PP@siRNA was dispensed onto the copper mesh in a 10-cm dish, with a layer of sealing film at the bottom. After 30 min, the remaining water was absorbed. The particles were stained with uranium acetate (Sigma-Aldrich, St. Louis, MO) for 15 min, followed by rinsing with water and absorbance of excess water. This process was repeated three times. Then the samples were allowed to dry and stored in a dedicated copper mesh container for observation with a transmission electron microscope (HT7800, Hitachi, Tokyo, Japan).

### Stability determination

Freshly prepared nanomedicine was dissolved in PBS and stored at 4°C, or dissolved in 20% fetal bovine serum (FBS) in PBS and stored in a 37°C incubator. Particle size was monitored for 7 days using DLS to assess its stability.

### Flow cytometry assay

bEnd.3 cells, Neuro-2a (N2a) cells, and BV-2 cells were seeded into 6-well plates at a density of 2 × 10^5^, 1 × 10^5^, and 5 × 10^4^ cells/well, respectively, cultured in DMEM (Gibco, Grand Island, NY) containing 10% FBS, and incubated in a 37°C incubator for 24 h. Then cell adherence was observed under a microscope. The culture medium was replaced with 2 mL of complete medium containing PBS, PPR@Cy5-siRNA, PP@Cy5-siRNA, or Free Cy5-siRNA (siRNA concentration set at 0.0012 μg/mL) under dark conditions, and incubated at 37°C for 3 h or 24 h. Then the medium was discarded, and cells were digested with trypsin (Gibco), and centrifuged at 1000 rpm for 3 min. Cells in the pellets were resuspended in 450 μL PBS, and then underwent flow cytometry (BD FACSAria ll, Franklin Lakes, NJ).

### Quantitative real-time PCR

N2a and BV-2 cells were plated in a 6-well plate at a density of 1×10^6^ and 1×10^5^ cells/well, respectively, cultured in 2 mL of complete culture medium for 24 h. Upon confirming cell adherence under a microscope, the culture medium was replaced with medium containing PPR@siBACE1, PP@siBACE1, free siBACE1, PBS, or Lipo2000@siBACE1. After incubation for three days, total RNA was extracted from the cells using extraction kits (TIANgen, Beijing, China). Total RNA was also extracted from the hippocampus and cortex tissues of mice. Reverse transcription kits (Yeasen, Shanghai, China) were then used to convert RNA into cDNA, and qRT-PCR (Applied Biosystems^TM^ 7500, Thermo Fisher Scientific) was performed to measure the mRNA expression level of *BACE1*.

### Cell imaging

N2a cells, bEnd.3 cells, and BV-2 cells were seeded onto cell slides in a 12-well plate at a density of 1×10^4^, 2×10^4^, and 5×10^3^ cells/well, respectively, and incubated at 37°C for 24 h. Then the culture medium was replaced with 2 mL of complete culture medium containing PBS, PPR@Cy5-siRNA, PP@Cy5-siRNA, or free Cy5-siRNA (siRNA concentration was 0.0012 μg/mL), and incubated for 3 or 24 h. Subsequently, the culture medium was replaced with medium containing the lysosomal dye Lysotracker green, and incubated at 37°C for 15 min in the dark. Then cells were fixed in 4% paraformaldehyde, and stained with DAPI (Beyotime, Shanghai, China). Cells were observed by super-resolution imaging microscopy (Elyra 7, Zeiss, Oberkochen, Germany).

### Western blot

Hippocampal/cortical tissues and treated N2a/BV-2 cells were homogenized in 500 μL PLB lysis buffer (Beyotime, Shanghai, China) with protease inhibitors, sonicated on ice, and centrifuged at 12,000 rpm for 20 min at 4°C. Supernatants were stored at − 80°C. Proteins were separated by SDS-PAGE, transferred to PVDF membranes, blocked, and incubated with primary antibodies and secondary antibody.

### Transcytosis evaluation in an in vitro BBB model

In vitro BBB model was established using Millicell hanging cell culture inserts (Merck, Darmstadt, Germany). In brief, bEnd.3 cells were seeded at a density of 8 × 10^4^ into the Transwell upper chambers pre-inserted into a 6-well plate. The cells were cultured continuously for 4 days with medium changes during this period. When the trans epithelial electrical resistances (TEER) value reached ~200 Ω*cm^2^, Cy5-siRNA, PP@Cy5-siRNA, or PPR@Cy5-siRNA was added to the upper chamber of the Transwell and incubated for 4 h. Then the nuclei of BV-2 cells and N2a cells in the lower chamber were stained with DAPI and observed under a Zeiss Elyra 7 microscope (Oberkochen, Germany). Cells in the lower chamber were resuspended in 200 μL PBS and underwent flow cytometry.

### Endocytosis inhibitor treatment

To elucidate the potential mechanism of cellular uptake of PPR@siRNA, endocytosis inhibitors, including methyl-β-cyclodextrin (M-β-CD, 5 mmol/L)(Selleck, Houston, TX), amiloride (Ami, 100 μmol/L) (Solarbio, Beijing, China), chlorpromazine (Chlo, 30 μmol/L) (Solarbio), genistein (Geni, 1 mmol/L) (Solarbio), and free RVG29 (500 and 100 μmol/L) (Top, Shanghai, China), were pre-incubated with bEnd.3, N2a, or BV-2 cells for 30 min, followed by incubation with Cy5-labeled PPR@siRNA. Untreated cells served as positive controls. After 4 h of incubation, cell uptake was assessed using flow cytometry.

### Biodistribution

Six WT mice were randomly divided into two groups (*n* = 3 per group) to receive injection of Cy5-labeled targeted nanomaterials (PPR-Cy5) or non-targeted nanomaterials (PP-Cy5) via the tail vein. After 2, 3, 4, 5, 6, and 8 h, the mice were anesthetized with isoflurane (Aladdin, Shanghai, China) (induction: 3%, maintenance: 1.5%), and in vivo imaging was performed. At 8 h post-injection, the mice were sacrificed, and perfusion was conducted. The intact brains, hearts, livers, spleens, lungs, and kidneys were removed, fixed in 4% paraformaldehyde for 24 h, and dehydrated in a gradient sucrose solution until sinking to the bottom. The tissues were embedded in tissue embedding medium O.C.T., and sectioned into 10 µm slices using a cryomicrotome. The resulting sections were stained with DAPI for nuclear staining. For mice receiving intravenous injection of PPR@Cy5-siRNA or PP@Cy5-siRNA (*n* = 3 per group), brain sections were stained for Iba1 and Nissl, while other organs were stained with DAPI. Fluorescence was observed using a digital scanning microscope (VS200, Olympus, Tokyo, Japan).

### Nanomedicine treatment in AD mice

Eight-month-old male FAD^4T^ mice were divided into four groups (*n* = 5) to receive intravenous injection of PPR@siBACE1, PP@siBACE1, free siBACE1, or PBS every other day at an equivalent dosage of 10 mg/kg PAMAM and 1000 µg/kg siRNA, for a total of 5 injections. The healthy control group consisted of WT mice.

### Open field test

Open field test was conducted to assess the anxiety state and locomotor ability of mice. The experimental apparatus consisted of a 50 cm × 50 cm × 50 cm open, white plastic square box. The box was divided into 16 square compartments. At the start of the experiment, a mouse was placed in the center of the box and allowed to explore for 10 min. The time spent in each compartment and the movement trajectories were recorded. The anxiety level and locomotor ability were evaluated by calculating the duration of stay in the central area and the overall movement distance.

### Nesting experiment

The nest construction experiment was performed to preliminarily evaluate hippocampal function and health status. Mice were individually housed in separate cages. Prior to testing, a 1-cm layer of crushed wood shavings was provided as bedding. On day 1, three soft 5 cm × 5 cm napkins were positioned centrally in each cage to assess nesting behavior. After 24 h, cages were photographed and scored as follows: 0, napkins unchanged; 1, napkins scattered without bite marks; 2, napkins concentrated centrally without bite marks; 3, napkins concentrated in one area with visible bite marks; and 4, majority of napkins bitten and piled together. Bite was identified as napkins fragmented or perforated.

### Novel object recognition

The novel object recognition apparatus was the same as that used for open field testing. One day prior to training, mice explored the empty chamber for 10 min. The task comprised training and testing phases. During training, mice were placed centrally with two identical objects in opposite corners, and allowed 5 min for exploration. After a 1-h interval, one object was replaced with a novel one for the 5-min testing phase. Exploration (sniffing or object manipulation, excluding proximity or passing) was quantified as the discrimination index as follows: discrimination index = (Time exploring novel object − Time exploring familiar object) / (Time exploring novel object + Time exploring familiar object).

### Morris water maze

Spatial learning and memory were assessed in a pool divided into four quadrants, each marked with distinct geometric symbols (pentagon, square, triangle, circle) as spatial cues. Water was added with food-grade titanium dioxide for tracking, and water temperature was maintained at 25 ± 2°C. In the training phase, mice underwent five consecutive days of training (four trials/day, 30-min inter-trial intervals). Each mouse was placed in the maze facing the wall of the pool, with a submerged platform randomly placed in one quadrant. The platform was submerged 0.5 cm below the water surface, making it invisible but reachable. The latency to locate the platform and the swimming paths were recorded. Mice that failed to find the platform within 60 s were guided to the platform and stayed there for 10 s. In the testing phase on day 6, the platform was removed. The mice were placed in the water, with the starting position being opposite to the target quadrant. The time spent in the target quadrant and platform-location crossings were recorded.

### Immunofluorescence staining of mouse brain sections

Brain sections underwent immunofluorescence staining using primary antibodies against Aβ (1:2000), Iba1 (1:1000), and GFAP (1:500), diluted in TBST containing 10% donkey serum. Stained sections were visualized under a digital scanning microscope (VS200, Olympus, Tokyo, Japan) to quantify Aβ plaque burden, microglial activation (Iba1), and astrocytic activation (GFAP).

### Measurement of Aβ_1-42_ content in mouse brain

Brain tissue was homogenized in PLB lysis buffer using a tissue grinder. Homogenates were centrifuged at 20,000× *g* for 30 min at 4°C, and supernatants were collected. Total protein concentration was determined by BCA assay. Aβ_1-42_ levels were quantified using a commercial ELISA kit (Elabscience, Wuhan, China).

### Circulating exosome isolation and measurement of exosomal Aβ_1−42_ content

Blood was collected from the orbital vein into EDTA-2Na-containing tubes, and centrifuged at 2200× *g* for 15 min at 4°C to isolate plasma. Plasma samples were stored at − 80°C within 1 h. After thawing, plasma samples were sequentially centrifuged at 2000× *g* for 30 min at 4°C to remove cells and debris, followed by centrifugation at 10000× *g* for 45 min at 4°C to remove larger microvesicles. The resulting supernatant was then ultracentrifuged at 120,000× *g* for 120 min at 4°C to pellet exosomes. The exosome pellet was washed once with PBS and re-pelleted by ultracentrifugation at 120000× g for 120 min at 4°C. The final purified exosome pellet was resuspended in an appropriate buffer for downstream analysis. Total exosomal protein was quantified by BCA assay, followed by Aβ_1-42_ measurement using an ELISA kit (Elabscience, Wuhan, China).

### BV-2 cell uptake of Aβ_1−42_ oligomers

BV-2 cells (3 × 10^4^ cells/well) were seeded in a 6-well plate and cultured for 24 h. Cells were incubated with Aβ_1-42_ oligomers (GenicBio, Shanghai, China) (2 μmol/L) alongside PBS, free siBACE1, PP@siBACE1, or PPR@siBACE1. After 24 h, cells were washed three times with PBS and observed under a Zeiss Elyra 7 microscope (Oberkochen, Germany).

### Hematoxylin and eosin staining

WT mice received five tail vein injections of PBS, PP, or PPR (PAMAM dose: 10 mg/kg). At 24 h post-final injection, mice were euthanized by cervical dislocation. Major organs (heart, liver, spleen, lung, kidney) were dissected, fixed in 4% paraformaldehyde for 24 h, dehydrated through an ethanol series (70%–100%), cleared in xylene, and paraffin-embedded. Sections (5 μm) were stained with hematoxylin (5 min), rinsed, differentiated in 1% acid ethanol, blued in ammonia water, counterstained with eosin (1 min), dehydrated, and cleared.

### Cell segmentation

Cell segmentation was performed using Cellpose3 (cyto3 model), leveraging lysosomal and nuclear positions to reconstruct cell membranes for precise identification.

### Statistical analysis

Data were processed using Origin 2021. Unless specified, results are expressed as mean ± standard deviation (SD). One-way ANOVA with Tukey’s post-hoc test was applied. *P* < 0.05 was considered as statistically significant.

## Results

### Characterization of PPR@siBACE1

**Fig. 2:**
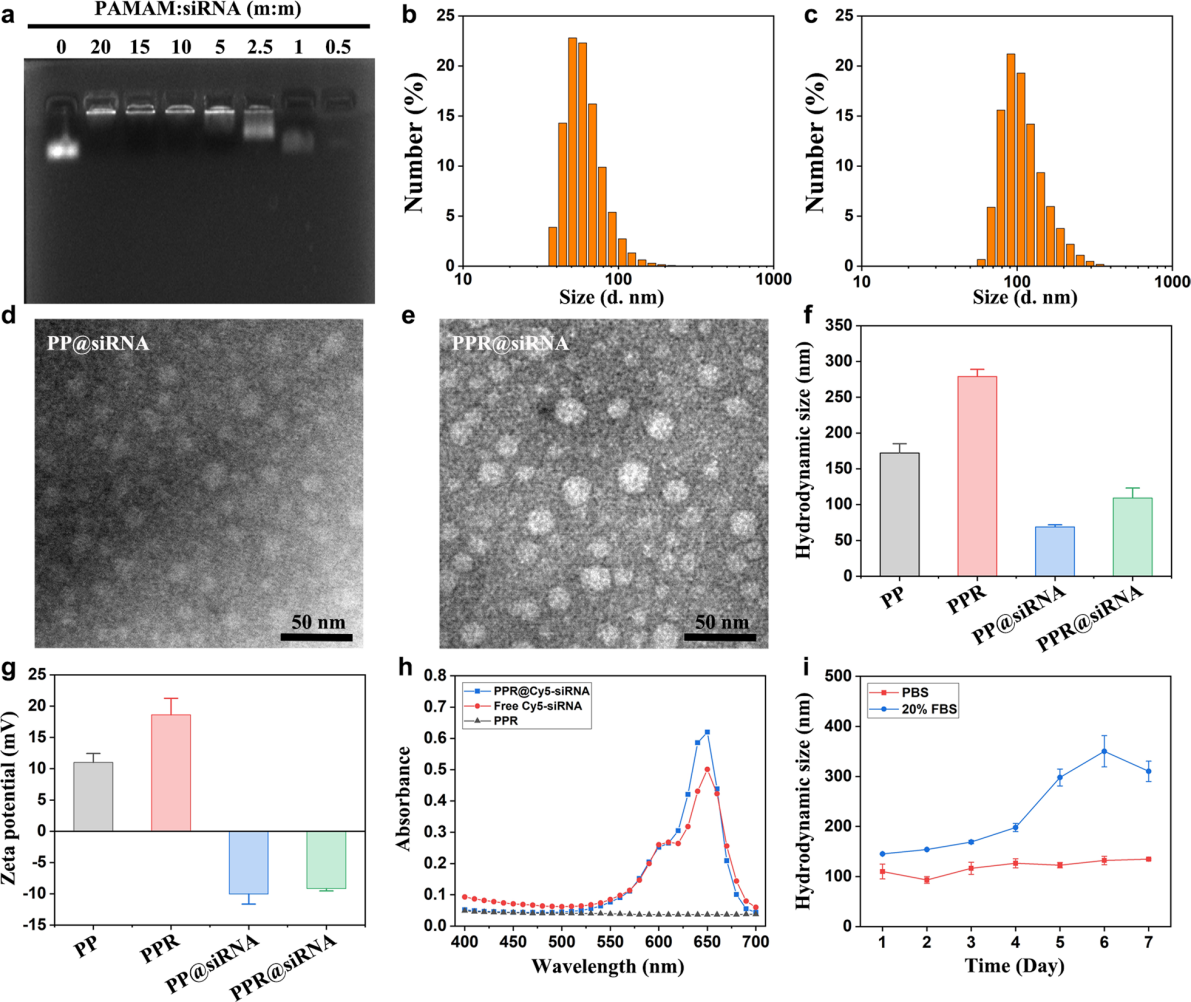
Characterization of PPR@siRNA. **a** Agarose gel electrophoresis of the nanomedicine produced by mixing siRNA with PPR at different mass ratios. **b**, **c** Size distribution by number of PP@siRNA (**b**) and PPR@siRNA (**c**) determined by DLS. **d**, **e** TEM images of PP@siRNA and PPR@siRNA. Scale bar, 50 nm. **f** Hydrodynamic sizes of PPR and PP before and after encapsulation of siRNA. **g** Zeta potential data of PPR and PP before and after encapsulation of siRNA. **h** UV–Vis spectrum of PPR, free Cy5-siRNA, and PPR@Cy5-siRNA. **i** Stability determination of PPR@siRNA in PBS and in 20% FBS in PBS for one week

Thiol-terminal RVG29 was reacted with maleimide-poly (ethylene glycol)-N hydroxysuccinimide (Mal-PEG-NHS) to obtain RVG29-PEG-NHS, which was then reacted with amino-enriched PAMAM to obtain RVG29-modified, PEGylated PAMAM (PPR). Meanwhile, amino-enriched PAMAM was reacted with methoxy-PEG-NHS to obtain PEGylated PAMAM (PP) as a control. PPR was confirmed by ^1^H nuclear magnetic resonance (^1^H NMR) (Fig. S1). Furthermore, agarose gel electrophoresis demonstrated that non-functional siRNA could be almost completely encapsulated at a low PAMAM-to-siRNA mass ratio of 5:1 after simply mixing PPR with negative control siRNA (Fig. 2a). Therefore, both PPR@siRNA and PP@siRNA were prepared at this mass ratio for subsequent studies. DLS analysis showed that the hydrodynamic size of PPR@siRNA and PP@siRNA were approximately 100 nm and 70 nm (Fig. 2b, c). TEM images demonstrated a spherical morphology with uniform dispersion of both PPR@siRNA and PP@siRNA (Fig. 2d, e). It should be noted that the actual diameter of PPR@siRNA was around 20 nm, which was much smaller than the diameter determined by DLS. This was primarily due to the abundant amino groups that PAMAM possesses, which can form a large hydration layer outside the PAMAM and thus increase the hydrodynamic size. Meanwhile, the electrostatic interaction between PAMAM and siRNA may result in a more compact structure, which well explains why the hydrodynamic sizes of PPR@siRNA and PP@siRNA were also smaller than PPR and PP, respectively (Fig. 2f).

Compared with PPR or PP, the zeta potential of PPR@siRNA or PP@siRNA was significantly reversed from positive charges to negative charges, suggesting successful loading of negatively charged siRNAs, which shielded the positively charged PAMAM (Fig. 2g). Moreover, the ultraviolet-visible (UV-Vis) spectrum of PPR loaded with Cy5-labeled siRNA (PPR@Cy5-siRNA) showed integrated characteristic peaks of both PPR and Cy5-siRNA (Fig. 2h). All these results underscore the siRNA-encapsulating ability of both PPR and PP. Additionally, PPR@siRNA stored in PBS for 1 week showed negligible changes of the hydrodynamic size (Fig. 2i). When stored in PBS containing 20% FBS, the hydrodynamic size showed a slight increase on day 4, and increased apparently from day 5. This suggests a good stability of PPR@siRNA, but potential protein absorption with longer incubation time which may compromise the stability of PPR@siRNA.

### Cellular uptake of PPR@siRNA

**Fig. 3:**
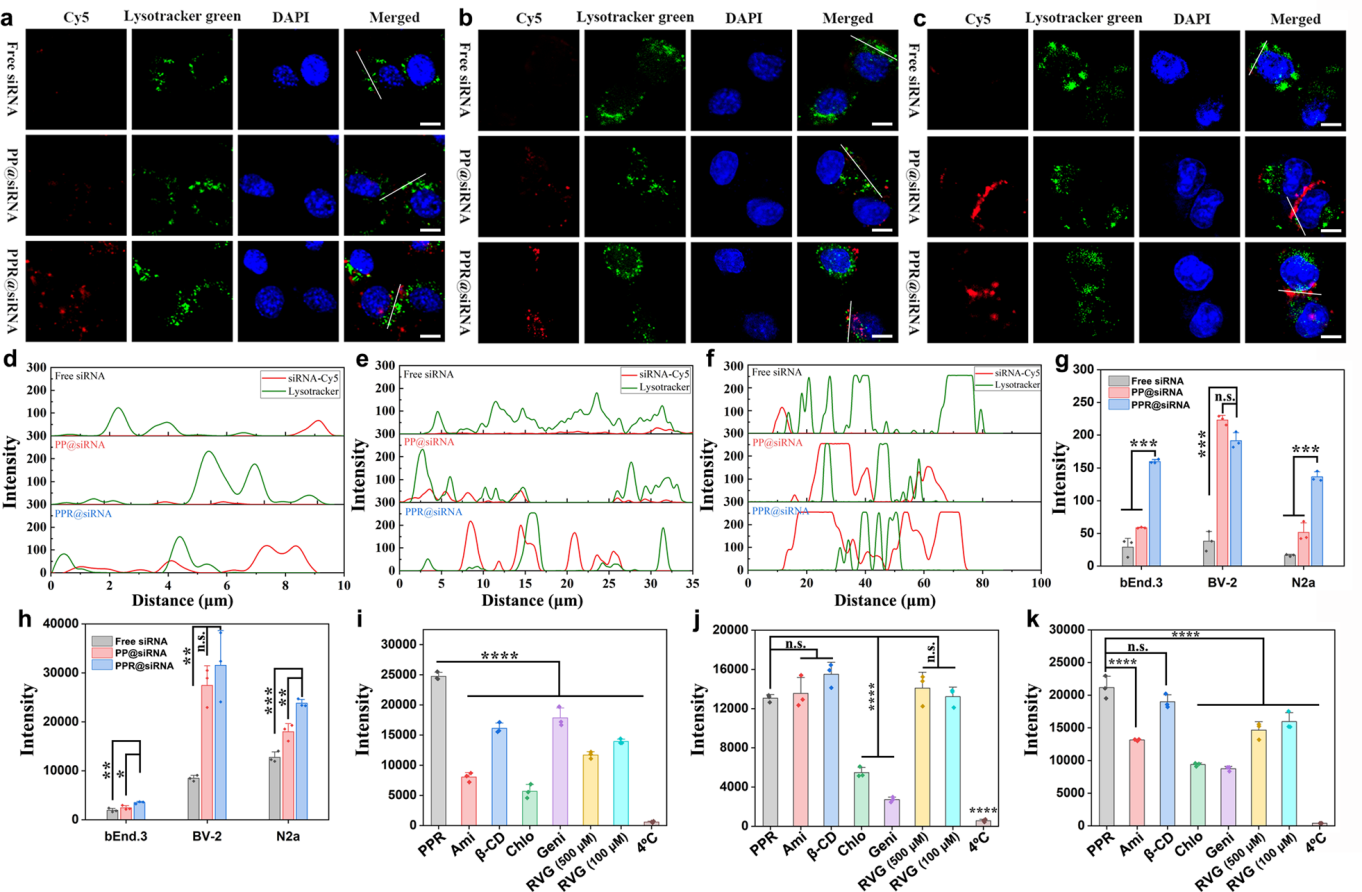
Cellular uptake of PPR@siRNA. **a**–**c** Representative confocal laser scanning microscopy images of bEnd.3 cells (**a**), N2a cells (**b**), BV-2 cells (**c**) incubated with free siRNA, PP@siRNA, and PPR@siRNA for 3 h. Scale bars, 5 μm. The analysis included ≥ 30 cells per group for confocal laser scanning microscopy. **d**–**f** Intensity curves along the indicative profile lines in bEnd.3 (**d**), N2a (**e**), BV-2 (**f**) cells. **g** Semi-quantitative fluorescence intensity (Cy5) of different formulations from microscopy images of (**a**–**c**). **h** Quantitative cellular uptake of different formulations by bEnd.3, N2a, and BV-2 cells at 24 h determined by flow cytometry. **i**–**k** Cellular uptake of PPR@siRNA by bEnd.3 (**i**), BV-2 (**j**), and N2a (**k**) cells at 6 h after pre-treatment with different inhibitors determined by flow cytometry. Mean ± SD, *n* = 3. *P < 0.05, **P < 0.01, ***P < 0.001, ****P < 0.0001

We next determined the cellular uptake of PPR@siRNA by bEnd.3 cells (a murine brain endothelial cell line), N2a (a murine brain neuroma cell line) and BV-2 (a murine microglia cell line), as well as the lysosome escape efficiency. Confocal laser scanning microscopy (CLSM) imaging showed a much stronger fluorescence signal of PPR@siRNA than that of PP@siRNA in bEnd.3 (Fig. 3a, Fig. S3) and N2a cells (Fig. 3b, Fig. S4) after 3-h incubation, indicating that RVG29 could facilitate cellular uptake by binding to nAChR, which is highly expressed on both cell lines. Moreover, the cellular uptake of free siRNA was significantly lower than both PPR@siRNA and PP@siRNA, mainly because the negatively charged siRNA was less efficient to cross the negatively charged cell membrane [38, 39]. However, the cellular uptake of PPR@siRNA by BV-2 was comparable to the uptake of PP@siRNA after incubation for 3 h (Fig. 3c, Figs. S2 and S5). This result may be attributed to the phagocytic nature of microglia, which possess a tendency to engulf foreign substances via a wide variety of scavenger receptors, Fcγ receptors, or complement receptors, instead of nAChR [40, 41]. All these observations were confirmed by semi-quantitative analysis of the fluorescence intensity in the three cell lines (Fig. 3g).

Furthermore, the poor co-localization between Cy5 and Lysotracker green in bEnd.3, N2a, and BV-2 cells suggested that both PPR@siRNA and PP@siRNA possess excellent lysosome escape capabilities (Fig. 3a–c). We next quantitatively analyzed the fluorescence overlay of Cy5-siRNA and Lysotracker-green. The intensity curves demonstrated poor overlay between Cy5-siRNA and Lysotracker green in bEnd.3, N2a, and BV-2 cells treated with PPR@siRNA or PP@siRNA (Fig. 3d–f). We attributed this capability to the proton sponge effect based on the buffering action of amino-enriched materials in a physiologically relevant range. The amino groups of PPR@siRNA or PP@siRNA could be chelated with protons provided by proton pump, leading to the continuous influx of Cl^−^ and water into lysosomes, causing swelling and rupture of lysosomal membrane and release of PPR@siRNA or PP@siRNA [42].

Next, we quantitatively assessed the cellular uptake of different formulations by the three cell lines using flow cytometry. Notably, bEnd.3 cells exhibited a robust capacity to internalize PPR@siRNA within 3 h, surpassing that of PP@siRNA and free siRNA by a significant margin (Fig. S6). At 24 h of incubation, although the uptake of PP@siRNA and free siRNA by bEnd.3 cells increased, it was still significantly lower than the uptake of PPR@siRNA (Fig. 3h). Similarly, N2a cells demonstrated a relatively superior ability to internalize PPR@siRNA compared to PP@siRNA. In line with the CLSM imaging, the uptake of PPR@siRNA and PP@siRNA by BV-2 cells was comparable, especially at 24 h, and both were much higher than the uptake of free siRNA. This further supports our hypothesis that BV-2 cells might internalize these nanomedicines via scavenger receptor-, Fcγ receptor-, or complement receptor-mediated phagocytosis, other than nAChR-mediated endocytosis.

To confirm our hypothesis, we next determined the underlying mechanisms involved in the internalization of PPR@siRNA. Before incubating with PPR@siRNA, cells were pre-treated with Ami, m-β-CD, Chlo, Geni or free RVG29, which inhibits micropinocytosis, lipid raft-mediated endocytosis, clathrin-mediated endocytosis, caveolin-mediated endocytosis, or nAChR-mediated endocytosis, respectively. In bEnd.3 cells, all inhibitors showed inhibiting effect on cellular uptake. Specifically, pre-treatment with Ami and Chlo resulted in 67.5% and 77.1% reduction of cellular uptake of PPR@siRNA compared with untreated condition, which was stronger than β-CD and Geni inhibition (Fig. 3i). This finding suggested that micropinocytosis and clathrin-mediated endocytosis are the predominant internalizing mechanisms in bEnd.3 cells. This is reasonable since receptor-mediated endocytosis also belongs to clathrin-mediated endocytosis. Moreover, pre-treatment with RVG29 also significantly reduced the uptake, mainly owing to the competitive binding with nAChR.

Similarly, in N2a cells, different inhibitors resulted in significant reductions of cellular uptake, except for β-CD. This indicated that N2a cells share similar internalizing mechanisms with bEnd.3 cells, through high expression of nAChRs (Fig. 3k). Unexpectedly, pre-treatment with Geni led to comparable reduction to Chlo, indicating that caveolin-mediated endocytosis might also play an important role in the internalization of PPR@siRNA by N2a cells.

By contrast, in BV-2 cells, only Chlo and Geni reduced the cellular uptake, while other inhibitors, particularly the free RVG29 peptide, showed negligible effect, suggesting the existence of a different internalization mechanism (Fig. 3j). This finding supports our previous hypothesis that the cellular uptake of PPR@siRNA by BV-2 was predominantly through receptor-mediated phagocytosis, instead of nAChR-mediated endocytosis. This is why Chlo showed inhibiting effect, whereas RVG29 did not affect the cellular uptake.

### Evaluation of BBB transcytosis in vitro and gene silencing efficiency

**Fig. 4:**
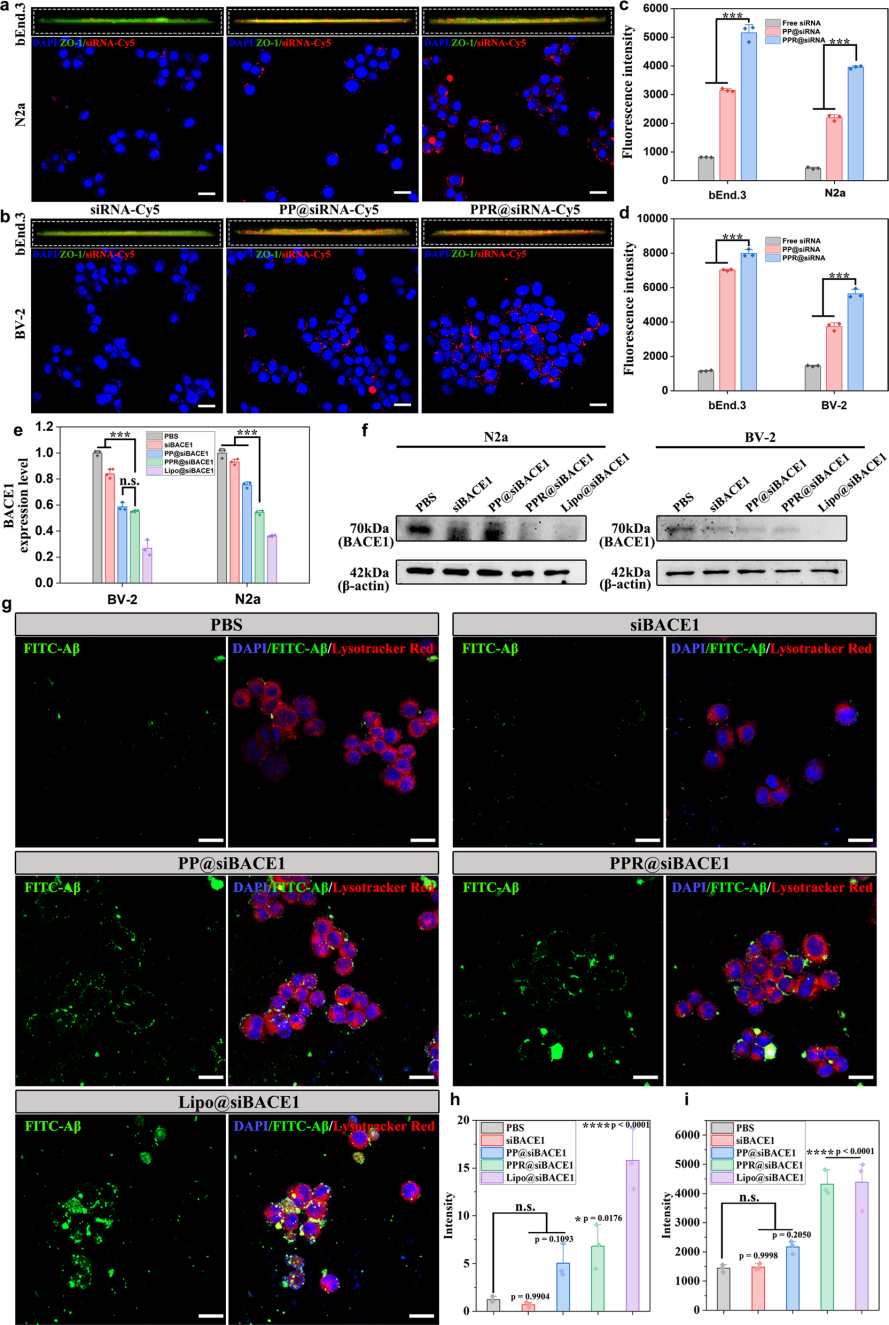
Evaluation of BBB transcytosis in vitro and gene silencing ability. **a, b** 3D construction of fluorescence signal from the bEnd.3 monolayer in top chamber and confocal laser scanning microscopy (CLSM) images of N2a cells (**a**) or BV-2 cells (**b**) in the bottom chamber, after treatment with different formulations. Scale bars, 20 μm. **c**, **d** Flow cytometry analysis of cellular uptake in the transwell model. Mean ± SD, *n* = 3. ***P < 0.001. **e** qRT-PCR analysis of *BACE1* mRNA expression in both N2a and BV-2 cells. Mean ± SD, *n* = 3. ***P < 0.001. **f** Western blots of BACE1 protein levels in N2a and BV-2 cells treated with different formulations. **g** CLSM images of BV-2 cells incubated with FITC-labeled Aβ for 4 h. The BV-2 cells were pre-treated with different formulations for 24 h. Scale bars, 20 μm. **h** Semi-quantification of fluorescence signal (FITC) from (**g**). Mean ± SD, *n* = 3. *P < 0.05, ****P < 0.0001 vs the PBS group. **i** Flow cytometry analysis of cellular uptake of Aβ in BV-2 cells after treatment with PBS, siBACE1, PP@siBACE1, PPR@siBACE1 or Lipo@siBACE1. mean ± SD, *n* = 3. ****P < 0.0001 vs the PBS group

To determine the BBB transcytosis efficiency of different formulations, we first established an in vitro BBB model by using a Transwell unit, where bEnd.3 cells were seeded in the top chamber and N2a or BV-2 cells were seeded in the bottom chamber. After introduction of Cy5-labeled formulations into the top chamber for 4 h, 3D fluorescence images showed low signal of free Cy5-siRNA overlayed with bEnd.3 monolayer, confirming that free siRNA was hard to cross BBB (Fig. 4a, b). By contrast, PPR@siRNA and PP@siRNA showed strong signals overlayed with the bEnd.3 monolayer. In addition, the signal of PPR@siRNA in either N2a or BV-2 in the bottom chamber was obviously stronger than PP@siRNA. Consistently, flow cytometry analysis further showed that PPR@siRNA had the highest cellular uptake by bEnd.3 in the top chamber and by N2a and BV-2 cells in the bottom chamber (Fig. 4c, d). All these results indicated that RVG29 modification not only enhanced the internalization by bEnd.3 monolayer, but also facilitated the transcytosis into the bottom chamber. Moreover, after transcytosis, PPR@siRNA still showed higher cellular uptake by N2a and BV-2 cells, suggesting the possibility to achieve dual targeting of neurons and microglia for gene silencing.

Given the enhanced cellular uptake, we subsequently encapsulated the functional siBACE1 and set out to investigate the gene silencing efficiency of PPR@siBACE1 in N2a and BV-2 cells. At 24 h post-transfection, qRT-PCR analysis showed that treatment with PP@siBACE1 reduced expression of *BACE1* mRNA in N2a cells compared to free siBACE1 treatment (Fig. 4e). In addition, treatment with PPR@siBACE1 led to a further decrease of *BACE1* mRNA expression compared with PP@siBACE1 and free siBACE1, which was higher than that with lipo2000@siBACE1 treatment, a commercial cationic liposome used for gene transfection. The enhanced gene silencing efficiency of PPR@siBACE1 was mainly due to its enhanced cellular uptake by N2a cells. The treatment with PPR@siBACE1 also significantly decreased expression of *BACE1* mRNA in BV-2 cells compared with free siBACE1 treatment, which was slightly lower than the PP@siBACE1 treatment (Fig. 4e). This result could also be explained by the fact that BV-2 cells could internalize PPR@siBACE1 and PP@siBACE1 in a nAChR-independent manner.

Consistently, western blotting analysis showed that PPR@siBACE1 significantly reduced BACE1 protein levels in N2a cells, with a much stronger knockdown effect compared to PP@siBACE1 and free siBACE1. In BV-2 cells, PPR@siBACE1 also reduced BACE1 expression, but the knockdown efficiency was less pronounced (Fig. 4f, Fig. S7). Meanwhile, PPR@siBACE1 was also comparable to lipo2000@siBACE1 treatment. Collectively, these results underscore the *BACE1* gene silencing capability of PPR@siBACE1 in both N2a and BV-2 cells compared to PP@siBACE1 and free siBACE1. As mentioned above, down-regulation of BACE1 expression in BV-2 cells may enhance their phagocytic capability towards Aβ. Therefore, we sought to validate the relationship between BACE1 expression and phagocytic capability. After pre-treatment with different formulations for 24 h, BV-2 cells were further incubated with fluorescein isothiocyanate (FITC)-labeled Aβ for another 4 h. CLSM images showed that BV-2 cells treated with free siBACE1 and PP@siBACE1 phagocytosed very limited Aβ, similar to the PBS-treated BV-2 cells (Fig. 4g, h, Fig. S8). Flow cytometry yielded consistent results (Fig. 4i). In sharp contrast, PPR@siBACE1-treated BV-2 cells phagocytosed a larger number of Aβ, although there was no significant difference between PPR@siBACE1 and PP@siBACE1.

### Evaluation of biodistribution in vivo

**Fig. 5:**
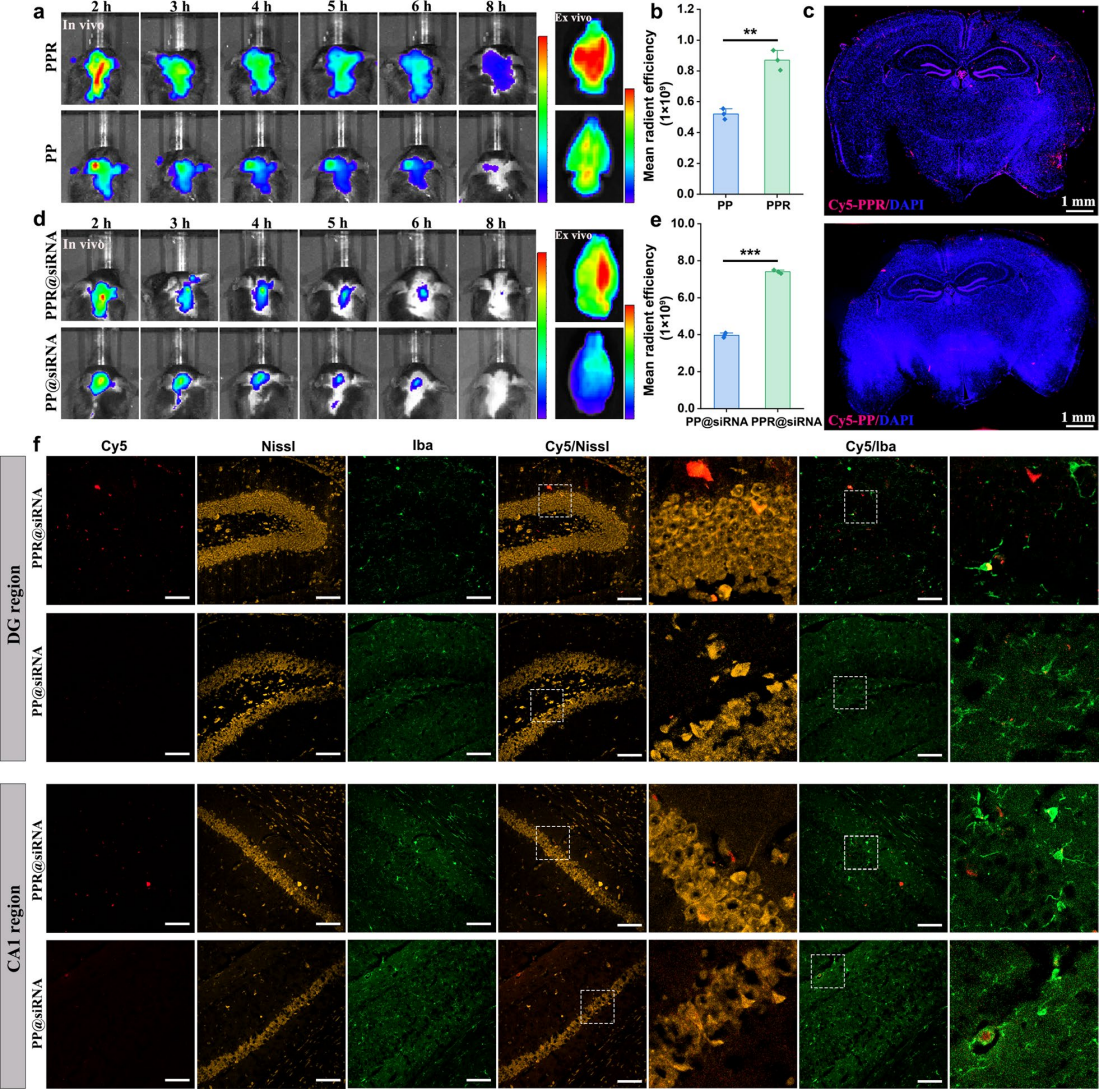
In vivo distribution and brain-targeting analysis. **a** In vivo imaging of Cy5-labeled PPR and PP in WT mice at different time intervals after intravenous injection, and ex vivo imaging at 8 h post-injection. In vivo bar: 0.5 × 10^9^–3.0 × 10^9^ ps^−1^ cm^−2^ sr^−1^/μW cm^−2^, ex vivo bar: 3.0 × 10^7^–5.0 × 10^7^ p s^−1^ cm^−2^ sr^−1^/μW cm^−2^. **b** Semi-quantification of fluorescence signals in (**a**) at 8 h. Mean ± SD, *n* = 3. **P < 0.01. **c** Digital scanning microscopy of brain sections from **a** at 8h. Scale bar, 1 mm. **d** In vivo imaging at different time intervals after intravenous injection with Cy5-labeled PPR@siRNA and PP@siRNA, and ex vivo imaging at 8 h post-injection. In vivo bar: 2.0 × 10^8^–4.0 × 10^8^ p s^−1^ cm^−2^ sr^−1^/μW cm^−2^, ex vivo bar: 2.0 × 10^8^–6.0 × 10^8^ p s^−1^ cm^−2^ sr^−1^/μW cm^−2^. **e** Semi-quantification of fluorescence signals in **d**. Means ± SD, *n* = 3. ***P < 0.001. **f** Cy5 fluorescence showing accumulation of Cy5-labeled PPR@siRNA and PP@siRNA in the DG and CA1 region of brain sections, which were also immune-stained for Nissl and Iba. Scale bars, 100 μm

To explore the brain-targeting and BBB transcytosis ability of nanocarriers, we next labeled the nanocarriers with Cy5 and monitored their biodistribution in WT mice using in vivo imaging system. At 2 h post-injection, in vivo fluorescence imaging displayed a stronger fluorescence signal of PPR in the brain than that of PP, indicating that RVG29 modification could navigate the nanocarriers to the brain site with high selectivity and promote transcytosis across the BBB (Fig. 5a, Fig. S9a). The fluorescence signals of PPR and PP groups gradually decreased, which was likely due to the rapid clearance following distribution. At 8 h post-injection, the fluorescence signal in the brain almost disappeared in the PP group, whereas the fluorescence signal of the PPR group still remained, suggesting that PPR had better brain retention.

The mouse brains were subjected to ex vivo imaging at 8 h post-injection. Quantitative analysis showed that the fluorescence intensity in the PPR group was about 1.8 folds of the PP group (Fig. 5b). These results further support the notion that PPR possessed better BBB transcytosis efficiency. To further explore the distribution site within the brain, we next observed fluorescence distribution in brain sections using a digital scanning microscope. Surprisingly, we found a much stronger fluorescence signal of PPR at the hippocampus, the main lesion area of AD, compared to that of PP (Fig. 5c). The enhanced accumulation of PPR within the hippocampus validated our design goal of increasing drug concentration at this region, although its cellular specificity for neurons versus microglia requires further investigation.

Next, we explored the in vivo distribution of nanocarriers loaded with Cy5-siRNA. As expected, both in vivo and ex vivo imaging showed that PPR@siRNA possessed stronger fluorescence signal at the brain site than PP@siRNA, confirming the contribution of RVG29 in brain-targeting delivery and BBB transcytosis (Fig. 5d, e and Fig. S9b). Likewise, CLSM images of brain sections demonstrated that PPR@siRNA accumulated more in the main pathological areas of AD, such as dentate gyrus (DG), CA1, and CA3 of the hippocampus, compared to PP@siRNA (Fig. 5f, Fig. S10–S13). More importantly, PPR@siRNA showed good co-localization with Nissl-labeled neurons and Iba1^+^ microglia. In contrast, PP@siRNA had relatively good co-localization only with microglia. Taking together, these results indicate that PPR@siRNA might be more effectively taken up by neurons via nAChR-mediated endocytosis and by microglia, while PP@siRNA was prone to be taken up by microglia instead of neurons. The co-localization of both PPR@siRNA and PP@siRNA with microglia can be explained by phagocytosis in a nAChR-independent manner.

### Evaluation of memory loss improvement

**Fig. 6:**
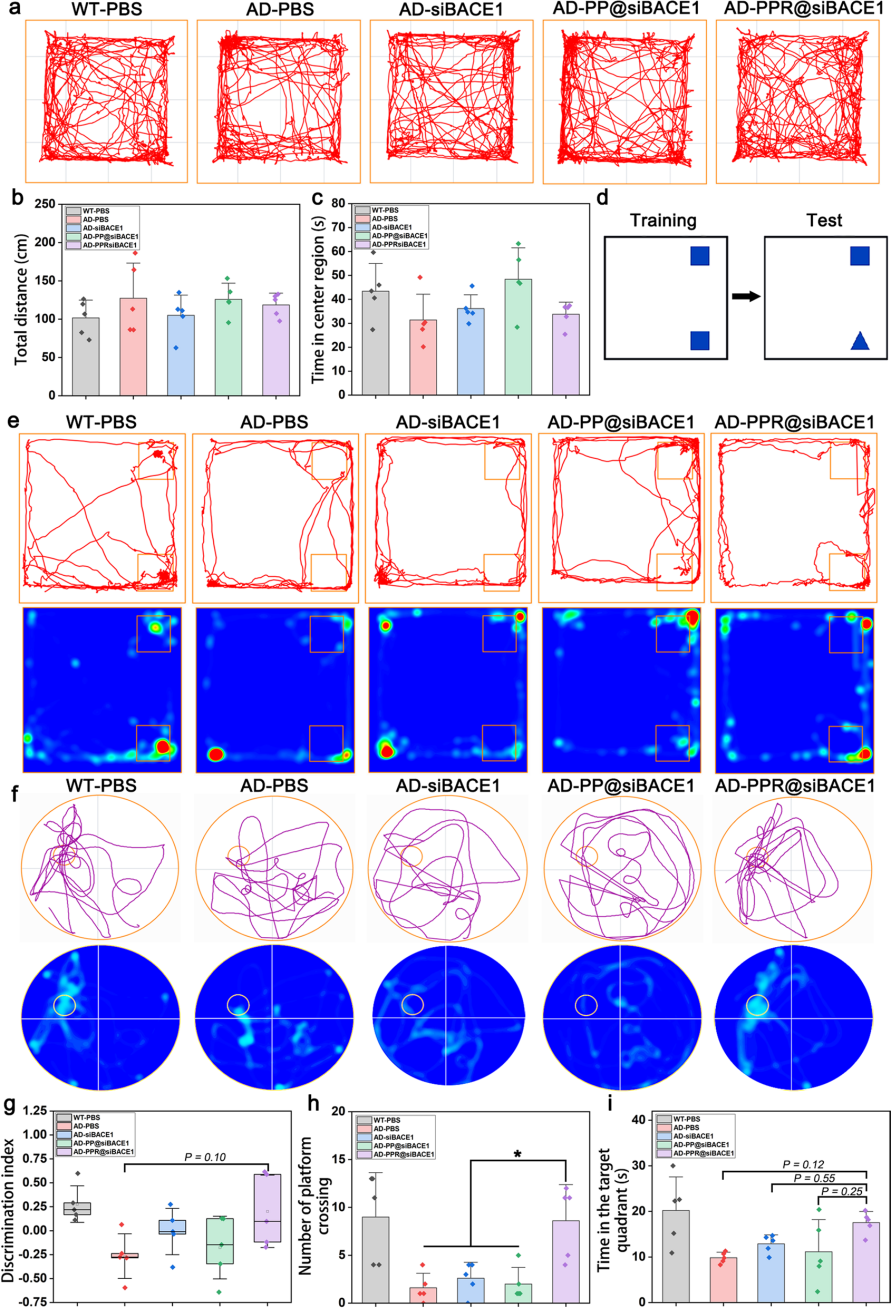
In vivo behavioral test. **a** Open field movement trajectories of mice in different groups. **b** Total distance traveled in the open field. **c** Time spent in the central region of the open field. Means ± SD, *n* = 5. **d**, **g** Diagram of novel object recognition setup and discrimination index. **e** Movement trajectories and heat maps of mice in different groups. **f** Swimming trajectories and heat maps in the water maze. **h**, **i** Number of platform crossings and time spent in the target quadrant. Data analyzed by one-way ANOVA with Tukey’s *post-hoc* test. Means ± SD, *n* = 5. *P < 0.05, **P < 0.01, ***P < 0.001

Based on promising results both in vitro and in vivo, we set out to assess the ability of PPR@siBACE1 to rescue memory decline in 8-month-old FAD^4T^ mice (Fig. S14a) after systemic administration for 5 doses. In the open field test, AD mice exhibited anxious behaviors, showing more movement in the peripheral regions of the open field, while the WT mice entered the central area more frequently (Fig. 6a). Quantitative analysis of the duration of stay in the center and the total distance traveled within the open field [43] showed no significant differences in either the time staying in the center or the total movement distance among the treatment groups (Fig. 6b, c). This suggested that PPR@siBACE1 had no significant effect on anxiety level and motor capability.

Nest-building is a natural daily life behavior in rodents, even when raised in laboratory settings [44]. In the nest-building test, WT mice were able to build nest successfully, while AD mice treated with PBS demonstrated less nest-building social collaboration skills. Treatment with PPR@siBACE1 improved nest-building performance. The nesting score in the PPR@siBACE1 treatment group was significantly higher than the PP@siBACE1 and siBACE1 groups, and comparable to the scores of WT mice treated with PBS (Fig. S14b, c). These findings suggest that PPR@siBACE1 treatment may have a beneficial impact on the hippocampal function and the overall health status of AD mice.

To confirm this claim, novel object recognition test was performed (Fig. 6d). The AD mice treated with PBS had less activities within the novel object region, whereas the WT mice treated with PBS exhibited much more activities (Fig. 6e). The AD mice treated with PPR@siBACE1 showed significantly increased activity trajectories across the region of novel object, which were comparable to the WT mice. In contrast, treatment with either PP@siBACE1 or free siBACE1 did not induce a noticeable change of the activity trajectories of AD mice (Fig. 6e). Analysis of discrimination index revealed decreased exploration of the novel object in AD mice treated with PBS compared to WT mice. However, AD mice treated with PPR@siBACE1 exhibited increased discrimination index, approaching levels comparable to WT mice. However, free siBACE1 or PP@siBACE1 did not improve the discrimination performance of AD mice (Fig. 6g).

Moreover, the Morris water maze test was performed to evaluate the spatial memory and learning ability of mice. After 5 days of training, the AD mice treated with PPR@siBACE1 exhibited a significantly shorter escape latency than other treatment groups (Fig. S14d). After removing the escape platform, the AD mice treated with PBS, siBACE1, or PP@siBACE1 displayed aimless trajectories in the water maze (Fig. 6f). In contrast, the PPR@siBACE1-treated AD mice had more travel trajectories in the target quadrant. Quantitative analysis showed significantly more crossings of the target platform and longer duration in the target quadrant in the PPR@siBACE1 group than the PP@siBACE1, free siBACE1, and PBS groups (Fig. 6h, i). These results verified the effectiveness of PPR@siBACE1 in rescuing the memory loss and improving cognitive ability of AD mice.

### Evaluation of gene silencing, Aβ clearance and neuroinflammation amelioration

**Fig. 7:**
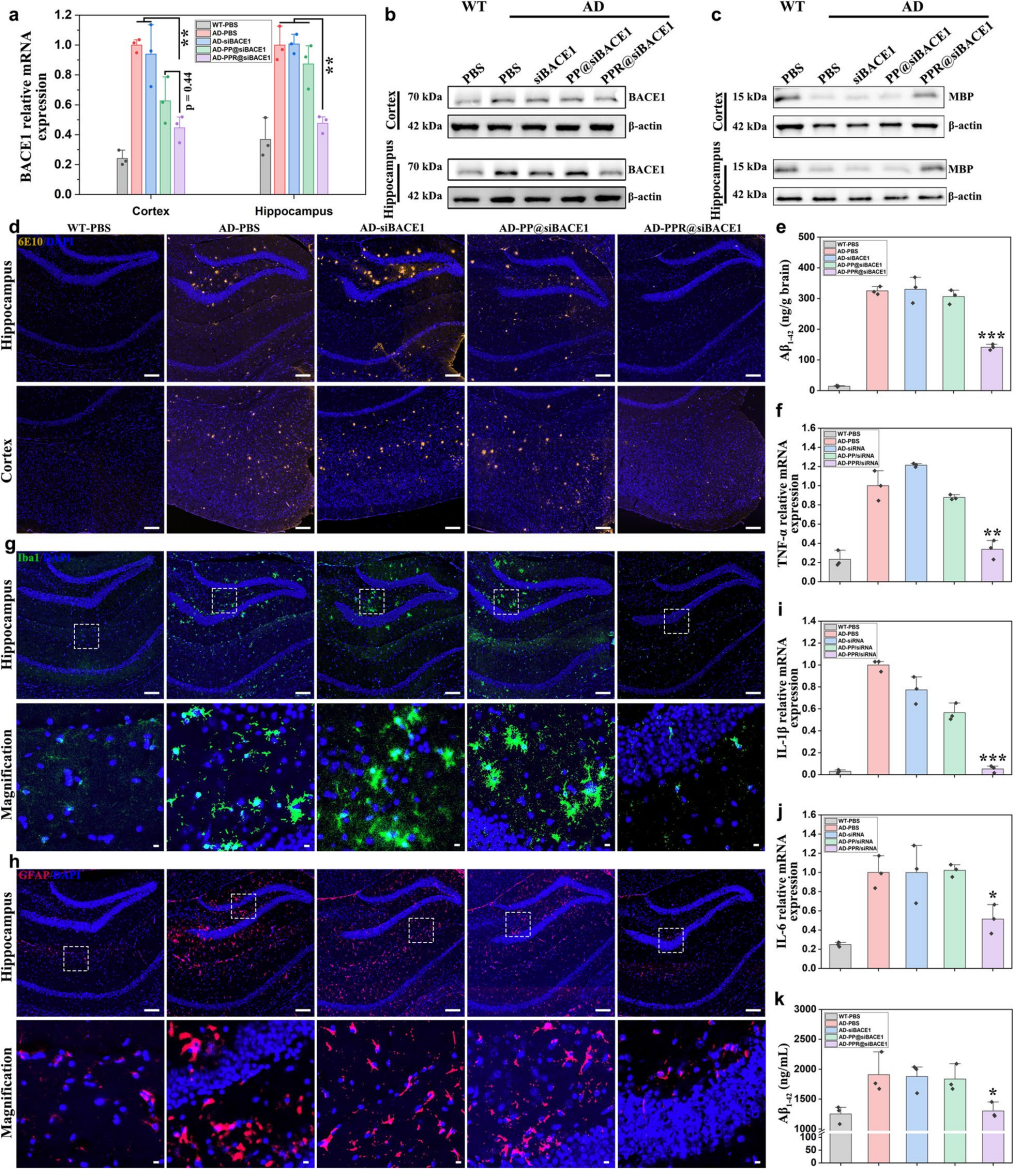
Therapeutic effects of PPR@siBACE1 in AD transgenic mice. **a** qRT-PCR analysis of *BACE1* mRNA expression in the hippocampus and cortex of AD mice treated with different formulations, and WT mice treated with PBS. **b** and **c** Representative western blots for BACE1 and MBP proteins in the hippocampus and cortex of AD mice treated with different formulations, and WT mice treated with PBS. **d** Immunofluorescence detection of Aβ plaques (yellow) in the hippocampus and cortex of AD and WT mice, with nuclei stained with DAPI (blue). Scale bars, 100 μm. **e** ELISA analysis of Aβ_1-42_ levels in the brains of mice from different groups. **g** Immunofluorescent staining of microglia with anti-Iba1 (green) in the hippocampus. Scale bars: 100 μm (original figure), 5 μm (magnified inset). **h** Immunofluorescent staining of astrocytes using anti-GFAP (red) in the hippocampus. Scale bars: 100 μm (original figure), 5 μm (magnified inset). **f**, **i**, **j** qRT-PCR analysis of the mRNA expression of inflammatory cytokines TNF-α (**f**), IL-1β (**i**), and IL-6 (**j**) in the brains of mice. **k** ELISA analysis of Aβ_1-42_ levels in circulating exosomes in the blood of mice. Mean ± SD, *n* = 3. *P < 0.05, **P < 0.01, ***P < 0.001 vs the AD-PBS group

To explore the mechanisms underlying the amelioration of memory loss and recognitive impairment, we next analyzed the effect of PPR@siBACE1 on gene silencing, Aβ clearance, and neuroinflammation. qRT-PCR analysis demonstrated a significant reduction of *BACE1* mRNA expression in both hippocampus and cortex of AD mice treated with PPR@siBACE1, in contrast to PBS, siBACE1, and PP@siBACE1 treatments (Fig. 7a). Furthermore, Western blotting analysis also revealed a notable decrease in BACE1 protein levels in both hippocampus and cortex of AD mice treated with PPR@siBACE1, compared to the PBS, siBACE1, and PPR@siBACE1 treatment (Fig.7b, Fig. S15a).

MBP, a major structural component of the axonal myelin sheath, has been reported to inhibit Aβ fibril formation and is deficient in patients with AD [45]. Compared with the PBS-treated WT mice, a significant reduction in MBP protein level was observed in the brains of PBS-treated AD mice (Fig. 7c, Fig. S15b). However, in PPR@siBACE1-treated AD mice, the MBP protein level was restored to levels comparable to those in PBS-treated WT mice. In PP@siBACE1- and siBACE1-treated AD mice, the MBP protein level was slightly higher than that in PBS-treated AD mice. These results indicated that knockdown of *BACE1* expression may reduce the requirement of MBP to neutralize Aβ_1−42_, thus leading to a high protein level of MBP, which indirectly supports the enhanced *BACE1*-silencing ability of PPR@siBACE1.

Immunofluorescence staining of brain sections revealed substantial deposition of Aβ plaques in the hippocampus and cortex of AD mice treated with PBS, whereas the PBS-treated WT mice exhibited negligible Aβ plaques (Fig. 7d). Treatment with PPR@siBACE1 resulted in a significant reduction of Aβ plaque deposition, which was mainly attributed to the reduced BACE1 expression and thus reduced Aβ production. In contrast, treatment with PP@siBACE1 or siBACE1 did not effectively reduce the Aβ plaque deposition. Furthermore, ELISA assay revealed that the Aβ_1−42_ content was reduced by 52% in the brains of PPR@siBACE1-treated AD mice compared to PBS-treated AD mice (Fig. 7e). However, the Aβ_1−42_ contents in the brains of PP@siBACE1- and siBACE1-treated AD mice were still comparable to that of the PBS group. Taking together, PPR@siBACE1 effectively alleviated Aβ burden in AD brains by silencing *BACE1* gene expression.

It has been reported that exosomes secreted by almost all eukaryotic cells, play a critical role in accelerating AD pathogenesis by spreading Aβ_1−42_ [46]. Therefore, we further determined the content of Aβ_1−42_ in circulating exosomes from peripheral blood of mice to see whether the treatment has an impact on circulating Aβ_1−42_. Surprisingly, ELISA analysis showed that treatment with PPR@siBACE1 significantly decreased the content of Aβ_1−42_ in exosomes isolated from blood of AD mice compared to PBS treatment (Fig. 7k). However, treatment with PP@siBACE1 and free siBACE1 did not induce any notable changes in the exosomal Aβ_1−42_ content compared to PBS treatment. We inferred that the Aβ_1−42_ content in circulating exosomes is closely relevant with Aβ_1−42_ production in the brain. Treatment with PPR@siBACE1 significantly reduces Aβ_1−42_ production, thus leading to reduced release of exosomal Aβ_1−42_ into the circulating blood. Although further studies are needed to validate this notion, the Aβ_1−42_ content in circulating exosomes could partly reflect the therapeutic effect of PPR@siBACE1.

Continuous microglial exposure to Aβ monomers, fibrils and plaques may promote their activation and phenotypic change towards the DAM-2 subtype, characterized by weak phagocytic ability and secretion of pro-inflammatory cytokines. To verify whether treatment with PPR@siBACE1 could reverse these effects of Aβ exposure, immunofluorescence staining of microglia was performed. Iba1 staining showed a large number of activated microglia in the hippocampus and cortex of AD mice treated with free siBACE1 and PP@siBACE1, similar to that of PBS-treated AD mice (Fig. 7g, Fig. S16). By contrast, the number of activated microglia was largely decreased in the AD mice treated with PPR@siBACE1, comparable to that of PBS-treated WT mice, suggesting that treatment with PPR@siBACE1 significantly reduced the activation of microglia.

Meanwhile, accumulating studies have reported that the activated microglia may also convert astrocytes from resting state to reactive state, generally surrounding the Aβ plaques to promote local inflammatory response [47]. Here, GFAP staining revealed a decrease of reactive astrocytes in the hippocampus and the cortex of AD mice treated with PPR@siBACE1 compared to AD mice treated with PP@siBACE1, free siBACE1, or PBS (Fig. 7h, Fig. S17). Moreover, qRT-PCR analysis showed that treatment with PPR@siBACE1 led to a remarkable reduction of mRNA expression of pro-inflammatory cytokines, including TNF-α, IL-1β, and IL-6, in the brains of AD mice compared to PP@siBACE1, free siBACE1, and PBS treatment (Fig. 7f, i, j). Collectively, these results indicate that PPR@siBACE1 treatment not only reduced activation of microglia and astrocytes, but also alleviated local neuroninflammation, primarily attributed to the reduced Aβ burden. Hematoxylin and eosin staining revealed that the nanomaterial used in this study did not induce necrosis or apoptosis in major organs following five doses, further confirming its safety profile (Fig. S18).

## Discussion

Aβ is a characteristic pathological hallmark and a key contributor to AD. Aβ is derived from the cleavage of APP by BACE1 (β-secretase) and γ-secretase, highlighting the significance of these enzymes in the disease process. Consequently, inhibiting the activity or expression of BACE1 in neurons offers a promising strategy to reduce Aβ production and thereby alleviate AD symptoms. However, although significant reductions of Aβ load have been observed in clinical trials, cognitive function improvements have remained elusive. One reason is the existence of additional pathogenic mechanisms beyond Aβ plaque deposition and tau protein phosphorylation. Notably, metabolic imbalances triggering microglial neuroinflammation are emerging as a critical factor in AD pathogenesis. Microglia, as resident immune cells of the brain, play a complex role in AD, initially clearing Aβ through phagocytosis to maintain brain homeostasis [48]. Nevertheless, excessive Aβ catabolism can trigger a microglial inflammatory storm, exacerbating Aβ deposition and neural damage [49]. Therefore, it is important to address the intricate "inflammatory vicious cycle" between abnormal Aβ metabolism and microglial dysfunction in AD brains [17]. Efficient clearance of Aβ, suppression of microglial inflammation, and controlling Aβ production at its source are crucial therapeutic strategies for effective AD treatment.

In this study, we present an easily prepared, reliable nanomedicine capable of directly targeting the disease site and specifically silencing *BACE1*. This nanomedicine could efficiently cross the BBB, reduce the expression of *BACE1* mRNA in neurons, thereby mitigating the production of Aβ. It also suppressed proinflammatory activation of microglia, mitigating neuroinflammation in AD.

In vivo distribution and immunofluorescence studies demonstrated that the developed PPR@siRNA can effectively penetrate the BBB and target neurons, predominantly mediated by nAChR, which is expressed on both brain capillary endothelial cells of BBB and neurons under physiological conditions. However, in the context of AD, the neuron-targeting efficiency may be compromised. The onset and progression of AD is characterized by a loss of neurons, particularly those expressing nAChRs [50]. Moreover, continuous exposure to toxic Aβ may impair the expression level of nAChRs in neuron [51]. Both can reduce the amount of nAChRs, challenging our design rationale and compromising the gene silencing efficacy. Therefore, PPR@siBACE1 administration at the right time before loss of neurons is critical for achieving satisfactory effects and clearance of pre-existing Aβ may also contribute to the delivery efficiency.

Although immunofluorescence staining also demonstrated that PPR@siRNA could be phagocytosed by microglia, the targeting specificity and mechanisms for microglia remain questionable. Previous studies revealed that nAChR is not only expressed on neurons, but also on microglia [52, 53]. This finding lends theoretical support to our design of also targeting microglia for gene silencing. However, our in vitro internalization study showed that pre-treatment with free RVG29 peptide had negligible influence on PPR@siRNA uptake by BV-2 cells. Therefore, we hypothesize an alternative internalization pathway, which is dependent on phagocytosis-related receptors, including scavenger receptors, Fcγ receptors, or complement receptors, instead of nAChRs. Further investigations are needed to elucidate the underlying mechanism for the targeted delivery of PPR@siRNA to microglia. Meanwhile, to achieve more precise and more efficient microglia-directed gene silencing, use of a ligand that can specifically target microglia is a promising strategy. For example, the MG1 peptide identified and isolated by the phage display technology has been reported to target microglia specifically [54]. Therefore, simultaneous targeted delivery to neurons and microglia can be achieved by either conjugating both RVG29 and MG1 peptides to a single delivery system or using separate delivery systems (each conjugated with either RVG29 or MG1 peptide separately). However, further investigation is required to determine which strategy is more effective and reliable.

BACE1 inhibition presents a promising therapeutic strategy for reducing amyloidogenesis. However, its translational application requires careful consideration of potential limitations associated with chronic inhibition. Failures in clinical trials (NCT02791191, NCT02569398, NCT01739348) suggest that prolonged BACE1 suppression may paradoxically exacerbate neurodegeneration, potentially resulting from impaired processing of BACE1 substrates such as neuregulin-1 and seizure protein 6 [55, 56]. These substrates play crucial roles in axonal guidance, synaptic maintenance, and myelination processes [57]. The observed clinical adverse effects, including worsening of cognitive decline in certain doses (NCT02791191), hepatoxicity (NCT02569398) and decreased hippocampal volumes (NCT01739348), underscore the need for optimized therapeutic strategies [58]. Future directions may include development of substrate-selective BACE1 modulators, intermittent dosing regimens, or combination therapies that preserve critical non-amyloidogenic functions while preventing pathological Aβ production [59, 60].

## Conclusions

In this study, we developed an RVG29-modified nanomedicine loaded with siBACE1 (PPR@siBACE1). PPR@siBACE1 demonstrated stability in vitro, efficient cellular uptake and lysosomal escape capability, efficient BBB penetration in vivo, and effective *BACE1* gene silencing. These findings suggest that PPR@siBACE1 holds significant therapeutic potential and clinical relevance for the treatment of AD.

## Supplementary Information

Additional file 1 (DOCX 10738 KB). **Supplementary Methods. Fig. S1.** 1H NMR spectra of synthesized PPR.** Fig. S2**. Cell segmentation of Fig. 2. **Fig. S3-S5.** Images for cellular uptake. **Fig. S6**. Quantitative cellular uptake of different formulations by bEnd.3, N2a, and BV-2 cells at 3 h determined from flow cytometry. **Fig. S7**. All Western blot of BACE1 protein expression levels in both N2a and BV-2 cells treated with different formulation. **Fig. S8**. Cell segmentation of Fig. 3. **Fig. S9**. In vivo imaging of WT mice treated with different formulation. **Fig. S10**. No Cy5 fluorescence signal detected in the DG region of brain sections of mice with PBS treatment. **Fig. S11**. No Cy5 fluorescence signal detected in the CA1 region of brain sections of mice with PBS treatment. **Fig. S12**. No Cy5 fluorescence signal detected in the CA3 region of brain sections of mice with PBS treatment. **Fig. S13**. Fluorescence determining the accumulation of Cy5-labeled PPR@siRNA and PP@siRNA in the CA3 region of brain sections. **Fig. S14**. Additional behavioral metrics. **Fig. S15**. All Western blot for BACE1 and MBP of mice. **Fig. S16**. Immunofluorescence staining of microglia with Iba1 (green) in the cortex region of mice from different groups. **Fig. S17**. Immunofluorescence staining of astrocyte with GFAP (red) in the cortex region of mice from different groups. **Fig. S18**. Representative data for hematoxylin and eosin staining in major organs of mice from different groups. **Table S1**. qPCR primer sequences

Additional file 2 (DOCX 7219 KB) Uncropped immunoblot images.

## Data Availability

All data are available in the main text or the supplementary materials.

## Abbreviations

APP: Amyloid precursor protein
AD: Alzheimer’s disease
BACE1: β-Site APP cleaving enzyme 1
Aβ: β-Amyloid
BBB: Blood brain barrier
PAMAM: Polyamidoamine
PEG: Polyethylene glycol
RVG29: Rabies virus protein 29
MBP: Myelin basic protein
DAM: Disease-associated microglia
