# Supplementary material for: Targeted delivery of BACE1 siRNA for synergistic treatment of Alzheimer's disease
Source: Transl Neurodegener. 2025 Aug 14;14:41. doi: 10.1186/s40035-025-00503-7 (PMC12351871; doi:10.1186/s40035-025-00503-7)
Supplement: Supplementary file 1 — Additional file 1 (DOCX 10738 KB). Supplementary Methods. Fig. S1. 1H NMR spectra of synthesized PPR. Fig. S2. Cell segmentation of Fig. 2. Fig. S3-S5. Images for cellular uptake. Fig. S6. Quantitative cellular uptake of different formulations by bEnd.3, N2a, and BV-2 cells at 3 h determined from flow cytometry. Fig. S7. All Western blot of BACE1 protein expression levels in both N2a and BV-2 cells treated with different formulation. Fig. S8. Cell segmentation of Fig. 3. Fig. S9. In vivo imaging of WT mice treated with different formulation. Fig. S10. No Cy5 fluorescence signal detected in the DG region of brain sections of mice with PBS treatment. Fig. S11. No Cy5 fluorescence signal detected in the CA1 region of brain sections of mice with PBS treatment. Fig. S12. No Cy5 fluorescence signal detected in the CA3 region of brain sections of mice with PBS treatment. Fig. S13. Fluorescence determining the accumulation of Cy5-labeled PPR@siRNA and PP@siRNA in the CA3 region of brain sections. Fig. S14. Additional behavioral metrics. Fig. S15. All Western blot for BACE1 and MBP of mice. Fig. S16. Immunofluorescence staining of microglia with Iba1 (green) in the cortex region of mice from different groups. Fig. S17. Immunofluorescence staining of astrocyte with GFAP (red) in the cortex region of mice from different groups. Fig. S18. Representative data for hematoxylin and eosin staining in major organs of mice from different groups. Table S1. qPCR primer sequences [file 40035_2025_503_MOESM1_ESM.docx]

Supplementary Materials for

**Targeted delivery of BACE1 siRNA for synergistic treatment of Alzheimer's disease**

Zhaohan Li^#^, Jun Yang^#^, Jianan Li, Shuxuan Zhao, Shaoping Jiang, Weimin Liu, Xinjian Li, Simeng Zhang, Haiyan Du, Junjun Ni, Yuanyu Huang^*^, Hong Qing^*^, Shaobo Ruan^*^

*Corresponding authors: Email: ruanshaobo@bit.edu.cn; hqing@bit.edu.cn; yyhuang@bit.edu.cn;

**Supplementary Methods**

**
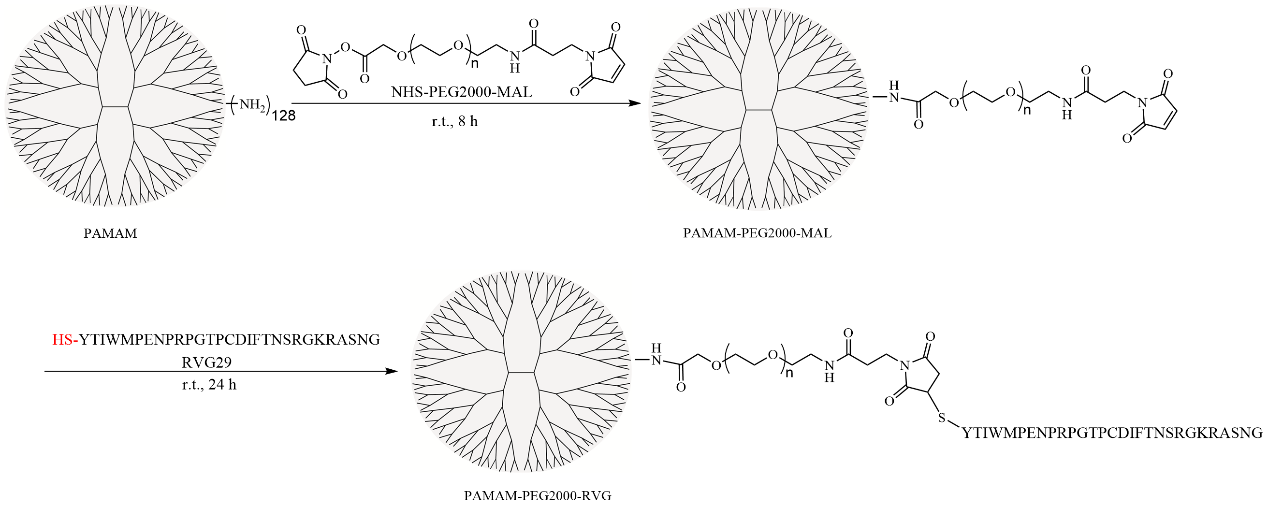
**

**Scheme S1**. Synthesis protocol of PPR

**Synthesis of PPR.** Dissolve COOH-PEG2000-MAL (Thanshtech, Guangzhou, China) (30 mg, 15 μmol), 1-(3-dimethylaminopropyl)-3-ethylcarbodiimide hydrochloride (EDC) (Meyer, Shanghai, China) (11.5 mg, 60 μmol), and N-hydroxysuccinimide (NHS) (Meyer, Shanghai, China) in Phosphate Buffered Saline (PBS) (6.903 mg, 60 μmol). Stir the solution at room temperature for 24 hours in a round-bottom flask. Subsequently, transfer an appropriate volume of polyamide-amine (PAMAM) (Macklin, Shanghai, China) methanol solution to a round-bottom flask and evaporate it to dryness using a rotary evaporator. Dissolve 43.2 mg (1.5 μmol) of PAMAM in PBS at a ratio of 1:10 (mol/mol) PAMAM to PEG. Mix with NHS-PEG-MAL and stir at room temperature for 8 hours. The NHS groups on NHS-PEG-MAL react specifically with the amino groups on the surface of PAMAM to synthesize PAMAM-PEG. Dialyze the reaction product against pure water using a dialysis bag with a molecular weight cutoff of 5000 Da for 24 hours, changing the water every 4 hours. Place the dialyzed product in a sample vial, remove the cap, wrap with parafilm, and then freeze-dry using a freeze-drying machine. Store the dried PAMAM-PEG in the refrigerator at -20℃.The synthetic targeted peptide RVG29 contains cysteine, whose sulfhydryl group (-SH) can specifically react with Maleimide (-MAL) to form a covalent linkage. Dissolve RVG29 (Top, Shanghai, China) (49 mg, 15 μmol) in PBS and mix it with a molar ratio of PAMAM:RVG29 = 1:10. Stir the mixture at room temperature for 24 hours to synthesize PAMAM-PEG-RVG29. The resulting product was then dialyzed against pure water using a dialysis bag with a molecular weight cutoff of 12000 Da for 24 hours, with the water changed every 4 hours. The dried PAMAM-PEG-RVG29 was stored in a refrigerator at -20°C.The solvent peak of CDCl_3_ is at 7.26 ppm and the characteristic absorption peak of methylene in the PEG structure is around 3.64 ppm. The backbone peak of PAMAM overlaps with RVG29 between 0.88-3.39 ppm. The multi-peak absorption peaks around 6.99 and 7.52 ppm are characteristic absorption peaks of the benzene ring in RVG29.

**UV-vis absorbance spectra.** The interaction of siRNA between PPR was investigated by UV-vis absorption spectra. The UV-vis absorption spectrum was taken from 400 to 700 nm.

**Cellular uptake assay.** In a 12-well plate, cell slides were placed into the wells, and N2a cells, bEnd.3 cells, and BV-2 cells were seeded into the wells (with a density of 1×10^4^ cells/well for N2a cells, 2×10^4^ cells/well for bEnd.3 cells, and 5×10^3^ cells/well for BV-2 cells). The 12-well plate was then placed into a 37°C incubator for 24 hours. After incubation, the culture medium was aspirated and discarded. Cell adhesion was observed under a microscope. The culture medium in the wells was aspirated and discarded, and 2 mL of complete culture medium containing PBS, PPR@Cy5-siRNA, PP@Cy5-siRNA, and Free Cy5-siRNA was added to each well, with a siRNA concentration of 0.0012 μg/mL. Incubation was performed at 37°C for 3 hours. Subsequently, the culture medium in the wells was aspirated and discarded and 4% paraformaldehyde was added to each well, and the paraformaldehyde was aspirated and discarded. Following fixation, cells were permeabilized with 0.1% Triton X-100 in PBS for 15 minutes at room temperature. Subsequently, actin filaments were labeled by incubating the samples with FITC-conjugated phalloidin (10 μg/mL in PBS, containing 1% dimethyl sulfoxide from the stock solution) for 30 minutes at room temperature. Unbound phalloidin (Abcam, Cambridge, UK) was removed by washing the samples three times with PBS. Afterwards, each well was stained with the nuclear dye DAPI (Beyotime, Shanghai, China) for 5 minutes, and the DAPI was aspirated and discarded. The cell slides were then removed with tweezers and mounted on glass slides coated with DAPI-containing anti-fluorescent quencher. The slides were sealed with nail polish. Observations and photography were conducted using super-resolution imaging microscopy (Elyra 7, Zeiss, Oberkochen, Germany).

**
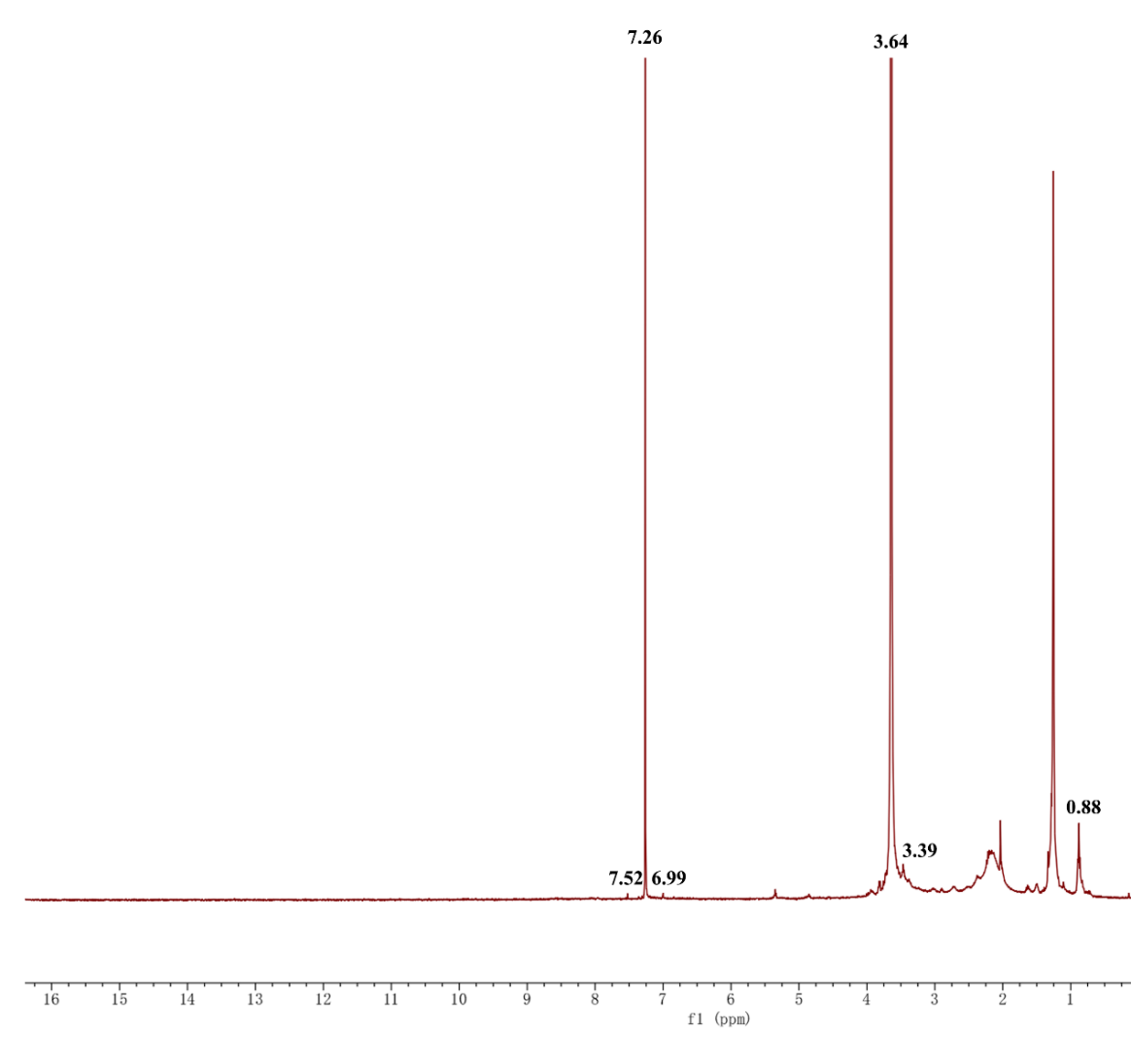
**

**Fig. S1.** ^1^H NMR spectra of synthesized PPR.


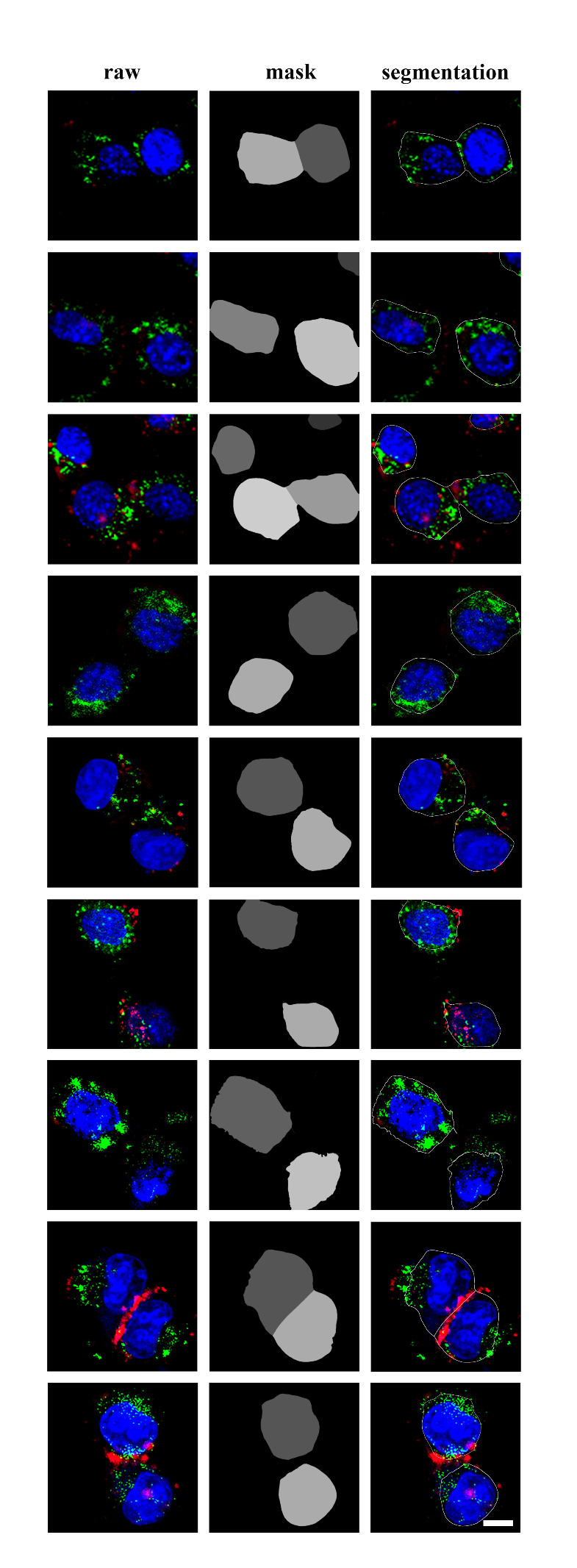


**Fig. S2.** Cell segmentation of Fig. 3a-c. Scale bar, 5 μm.


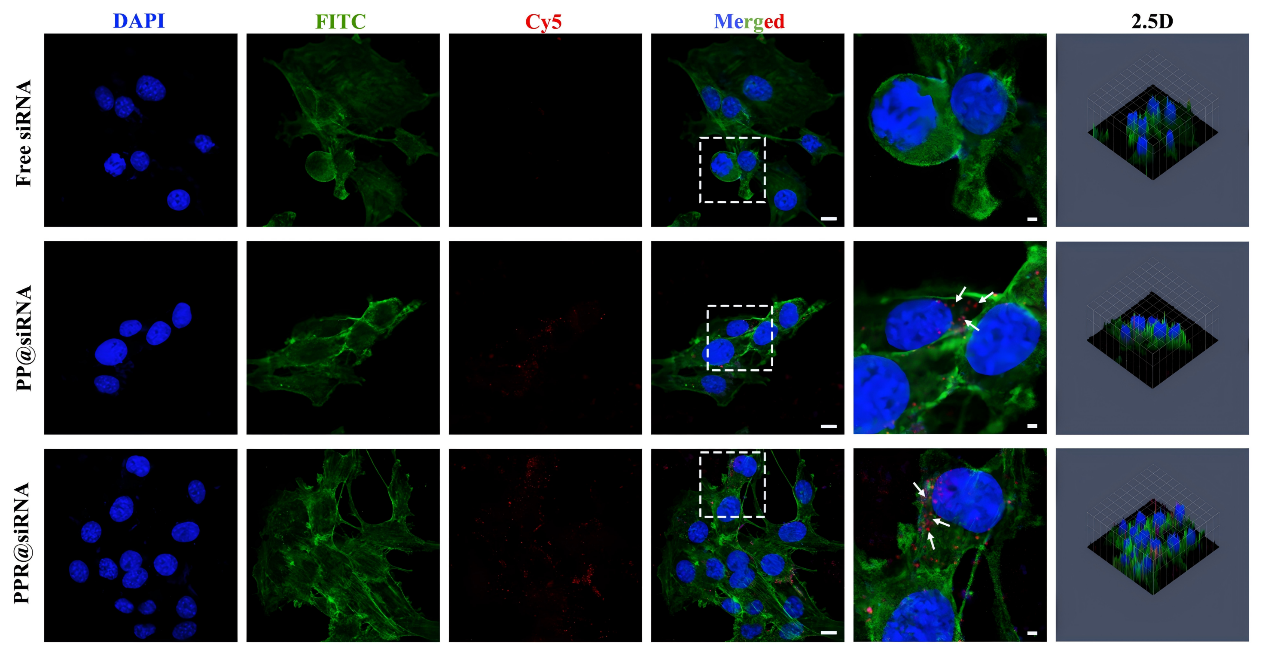


**Fig. S3.** Images for cellular uptake. Images were collected for bEnd.3 cells. Cell nuclei were stained with DAPI (blue), siRNA was labeled by Cy5 dye (red), and cell cytoskeleton was stained with FITC-phalloidin (green) to indicate cytoplasm area. Scale bars: 5 μm (original figure), 1 μm (magnified inset).


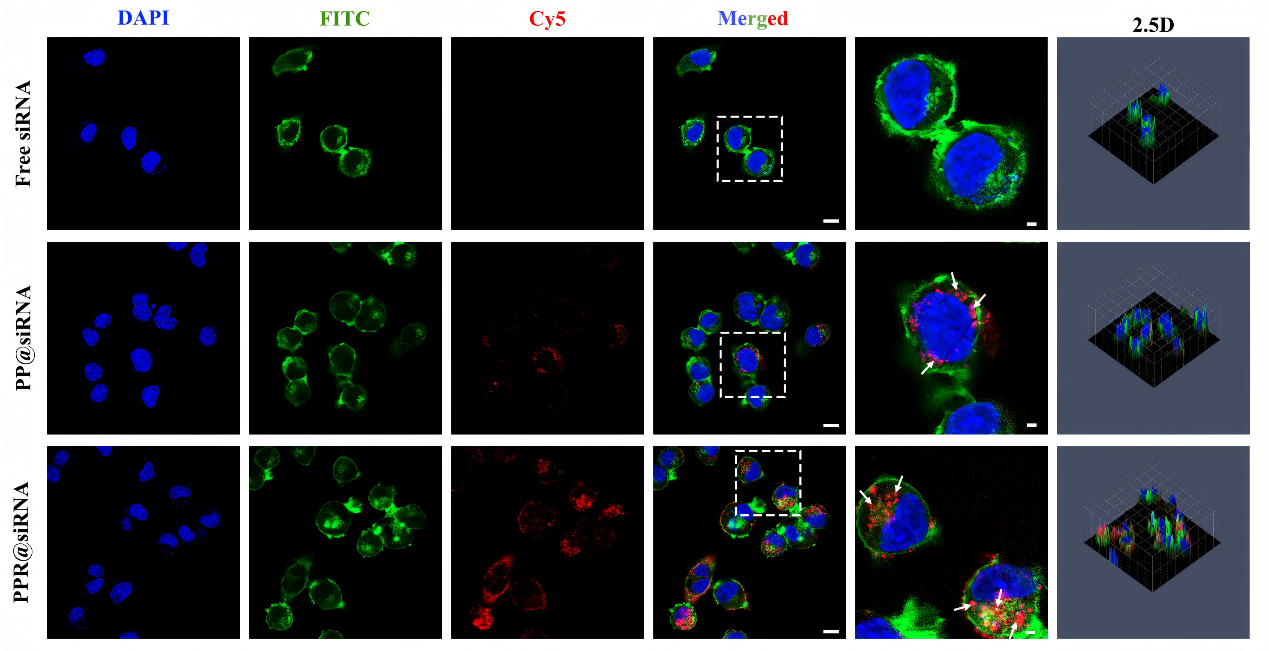


**Fig. S4.** Images for cellular uptake. Images were collected for N2a cells. Cell nuclei were stained with DAPI (blue), siRNA was labeled by Cy5 dye (red), and cell cytoskeleton was stained with FITC-phalloidin (green) to indicate cytoplasm area. Scale bars: 5 μm (original figure), 1 μm (magnified inset).


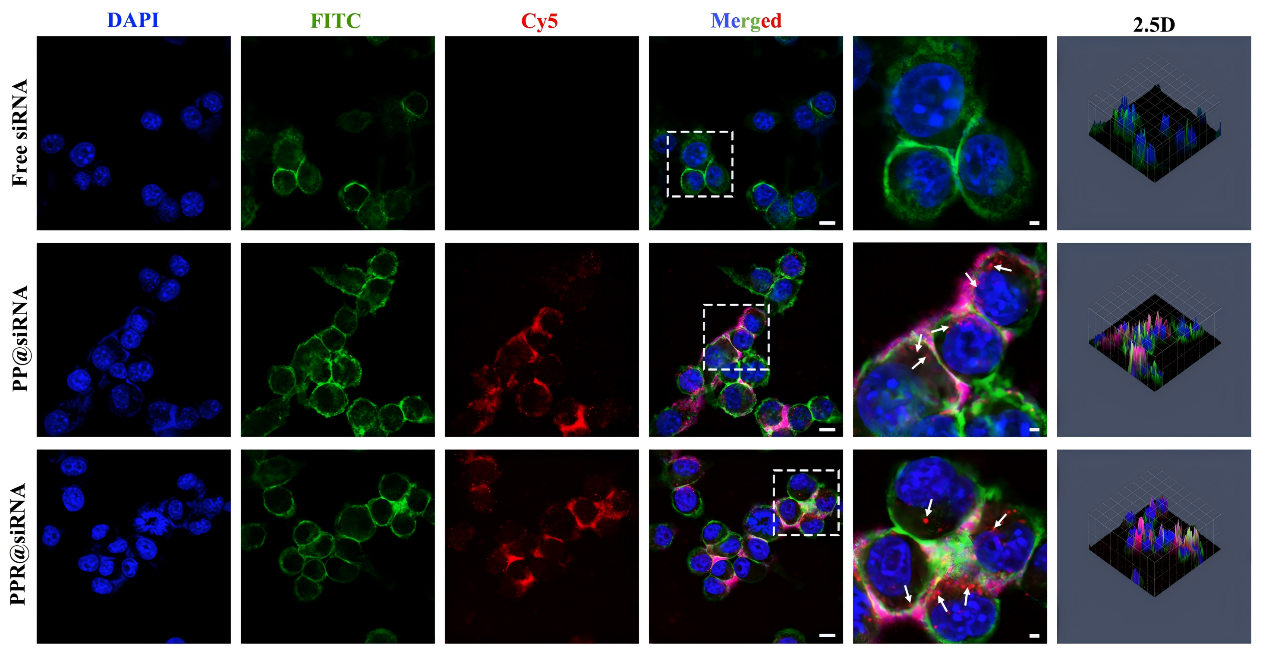


**Fig. S5.** Images for cellular uptake. Images were collected for BV-2 cells. Cell nuclei were stained with DAPI (blue), siRNA was labeled by Cy5 dye (red), and cell cytoskeleton was stained with FITC-phalloidin (green) to indicate cytoplasm area. Scale bars: 5 μm (original figure), 1 μm (magnified inset).


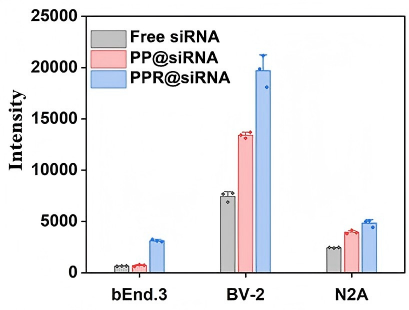


**Fig. S6.** Quantitative cellular uptake of different formulations by bEnd.3, N2a, and BV-2 cells at 3 h determined from flow cytometry.


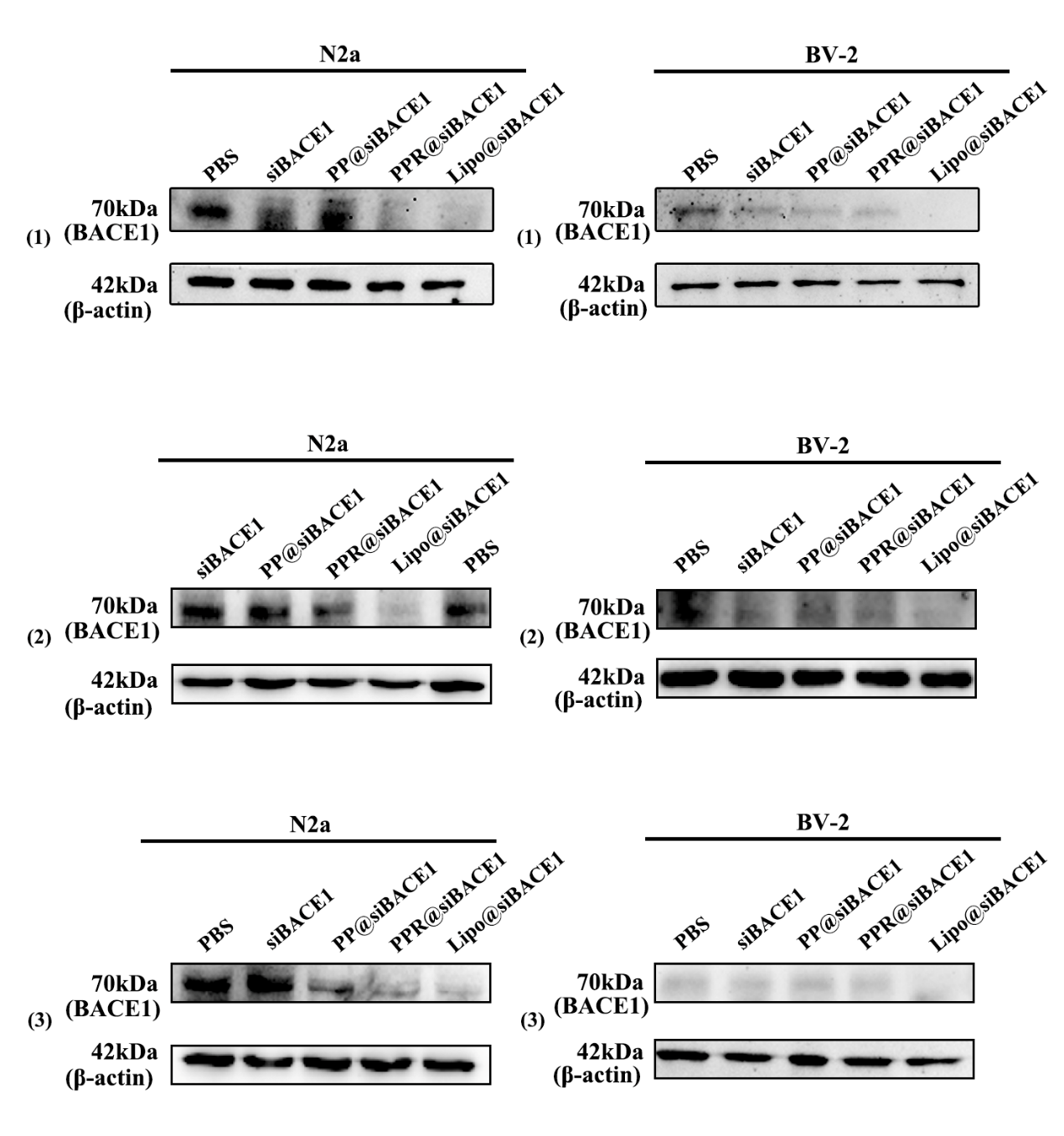


**Fig. S7.** All Western blot of BACE1 protein expression levels in both N2a and BV-2 cells treated with different formulation.


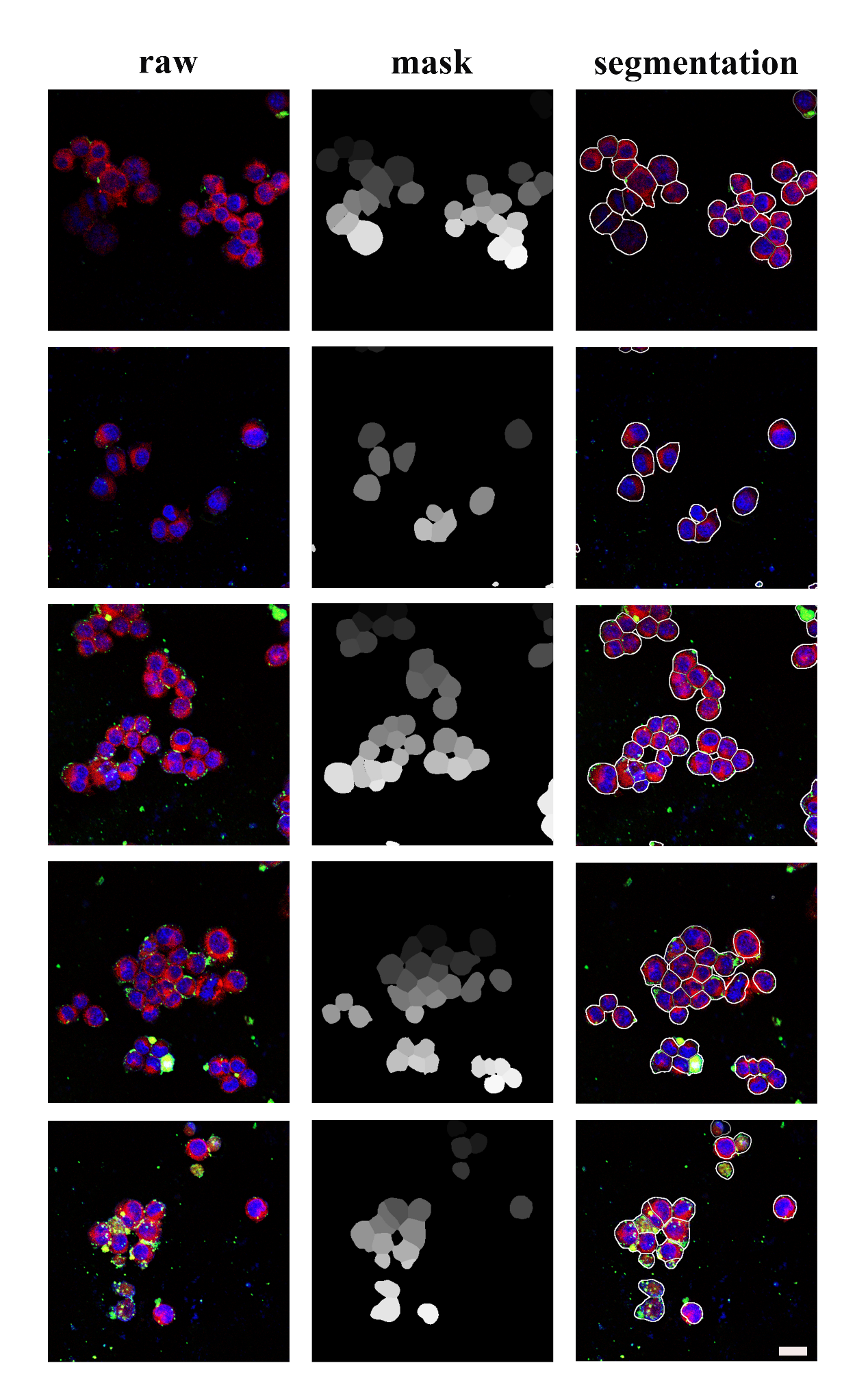


**Fig. S8.** Cell segmentation of Fig. 4g. Scale bar, 20 μm.


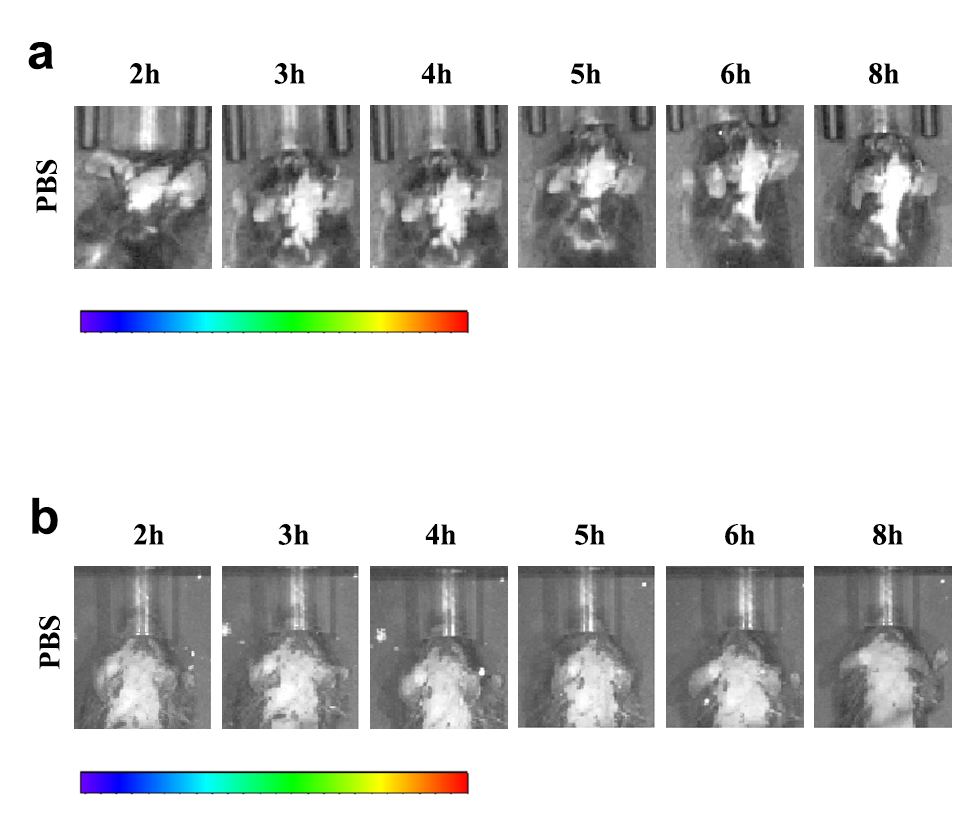
**Fig. S9.** In vivo imaging of WT mice treated with different formulation. (**a**) In vivo imaging of WT mice after intravenous injection with PBS as control for Cy5-labeled PPR and PP at different time intervals, bar: 0.5×10^9^-3.0×10^9^ [p s^−1^ cm^−2^ sr^−1^ ]/[μW cm^−2^]. (**b**) In vivo imaging of WT mice after intravenous injection with PBS as control for Cy5-labeled PPR@siRNA and PP@siRNA at different time intervals, bar: 2.0×10^8^-4.0×10^8^ [p s^−1^ cm^−2^ sr^−1^ ]/[μW cm^−2^].


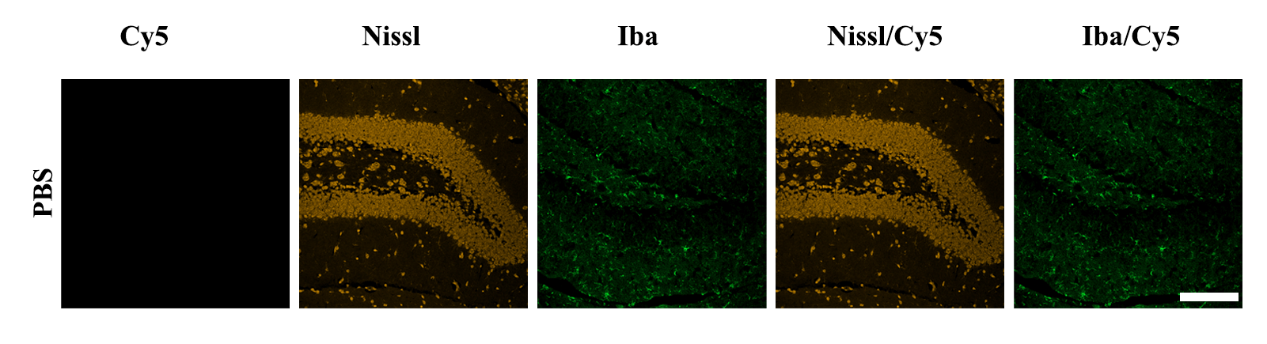


**Fig. S10.** Fluorescence determining the accumulation of PBS in the DG region of brain section where was immune-staining with Nissl and Iba. Scale bar, 100 μm.


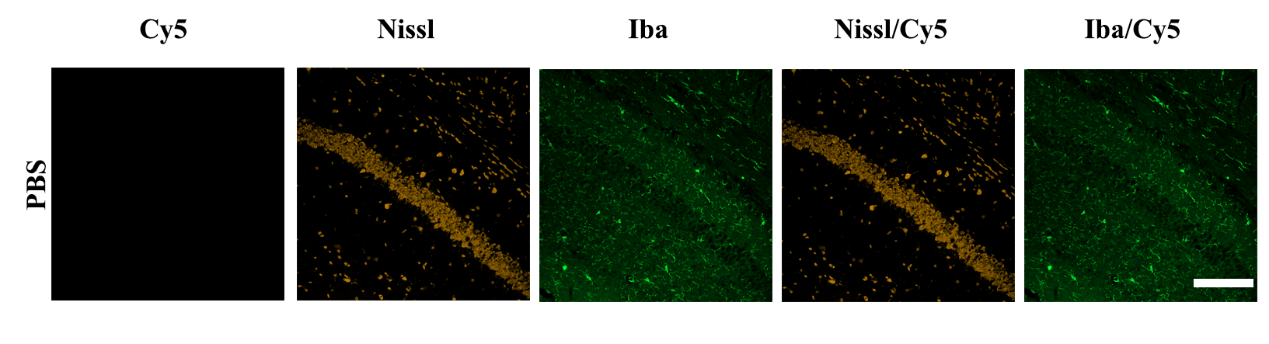


**Fig. S11.** Fluorescence determining the accumulation of PBS in the CA1 region of brain section where was immune-staining with Nissl and Iba. Scale bar, 100 μm.


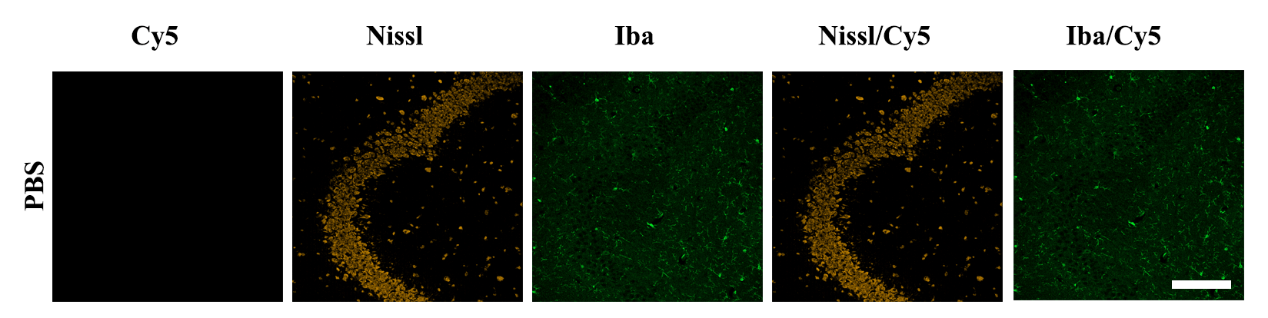


**Fig. S12.** Fluorescence determining the accumulation of PBS in the CA3 region of brain section where was immune-staining with Nissl and Iba. Scale bar, 100 μm.

**
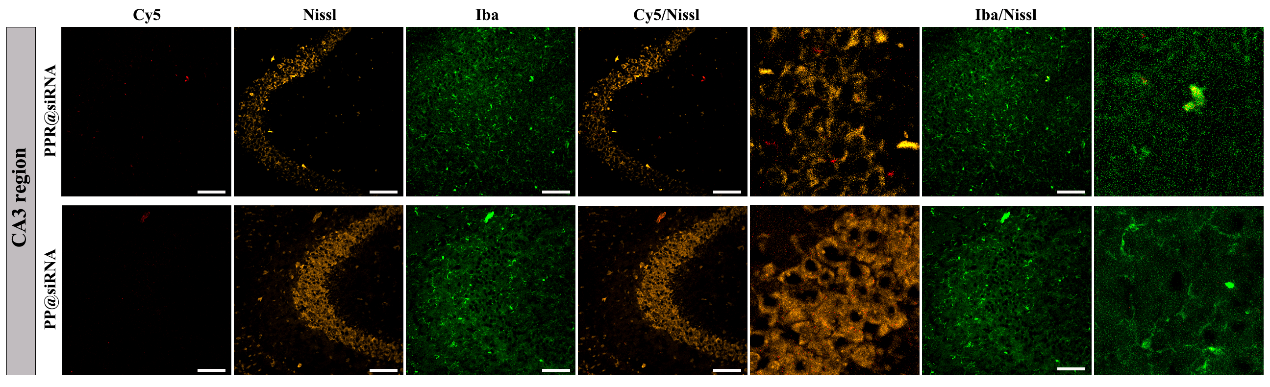
**

**Fig. S13.** Fluorescence determining the accumulation of Cy5-labeled PPR@siRNA and PP@siRNA in the CA3 region of brain section where was immune-staining with Nissl and Iba. Scale bar, 100 μm.


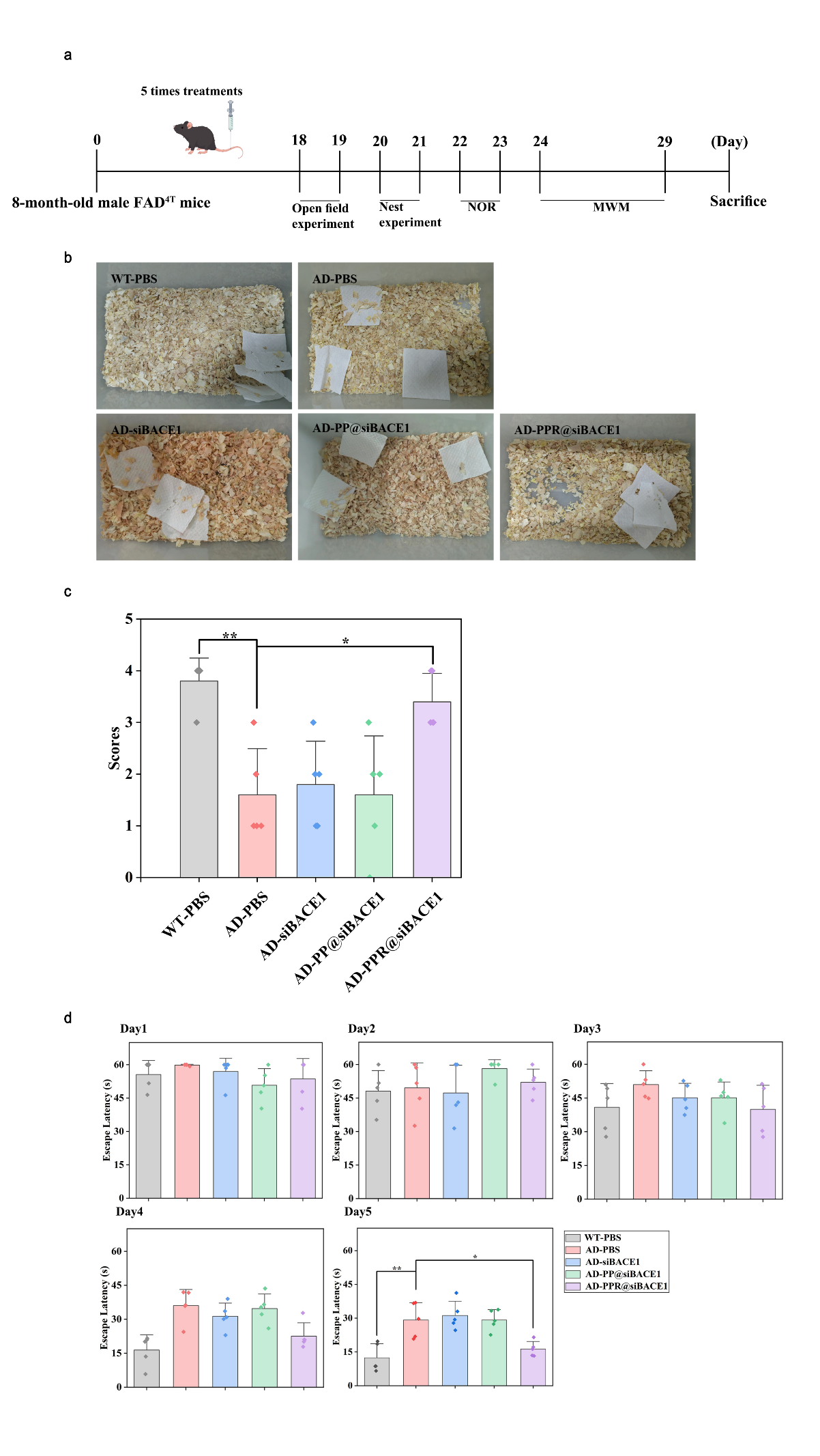


**Fig. S14.** Additional behavioral metrics. **(a)** Time course of administration and therapeutic evaluation. (**b)** Pictures of mouse nesting situation. (**c)** Nesting experiment scores of different groups of mice. Graph: Mean ± SD, *n* = 5. **P* < 0.05, ***P* < 0.01. (**d)** MWM escape latencies in different groups of mice. Graphs: Mean ± SD, *n* = 5. **P* < 0.05, ***P* < 0.01.

**
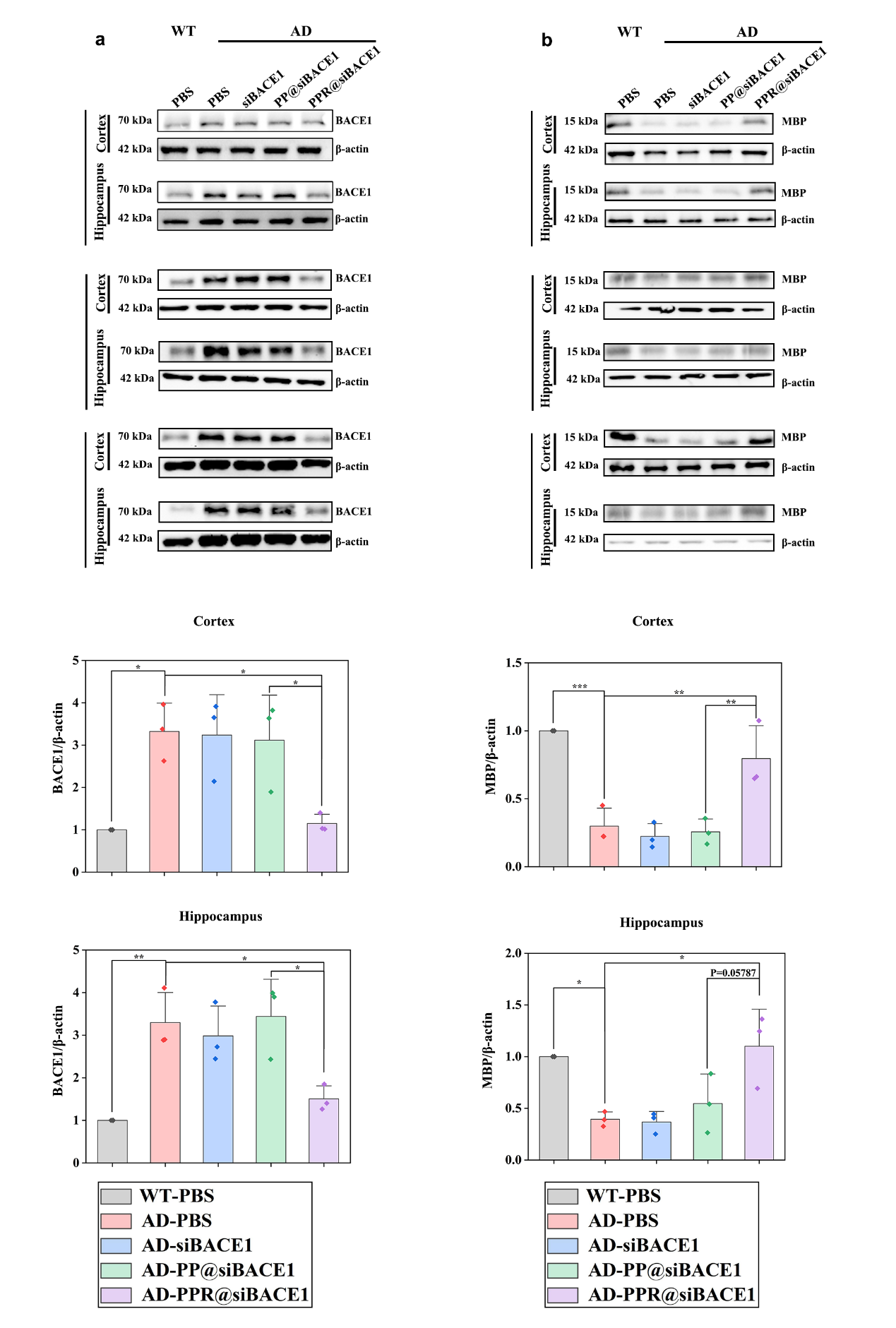
** **Fig. S15.** All Western blot for BACE1 and MBP of mice. **(a)** Western blot data for BACE1 protein expression in hippocampus and cortex region of AD mice treated with different formulations, and WT mice treated with PBS were served as control. **(b)** Western blot data for MBP protein expression in hippocampus and cortex region of AD mice treated with different formulations, and WT mice treated with PBS were served as control. Graphs: Mean ± SD, *n* = 3. **P* < 0.05, ***P* < 0.01, ****P* < 0.001.


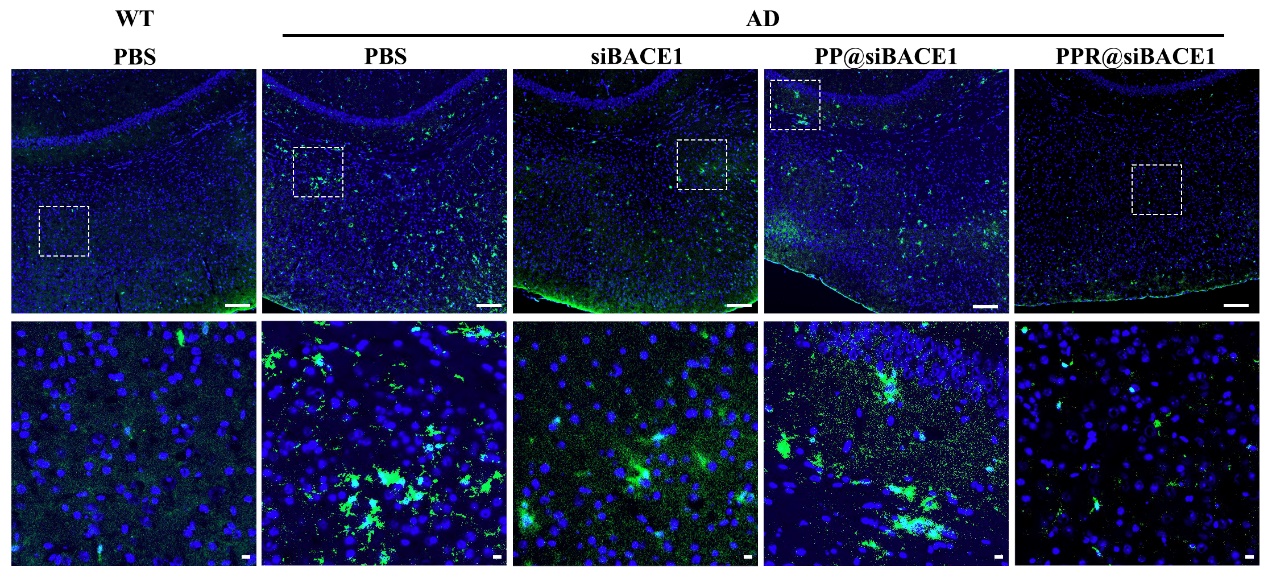


**Fig. S16.** Immunofluorescence staining of microglia with Iba1 (green) in the cortex region of mice from different groups. Scale bars: 100 μm (original figure), 5 μm (magnified inset).


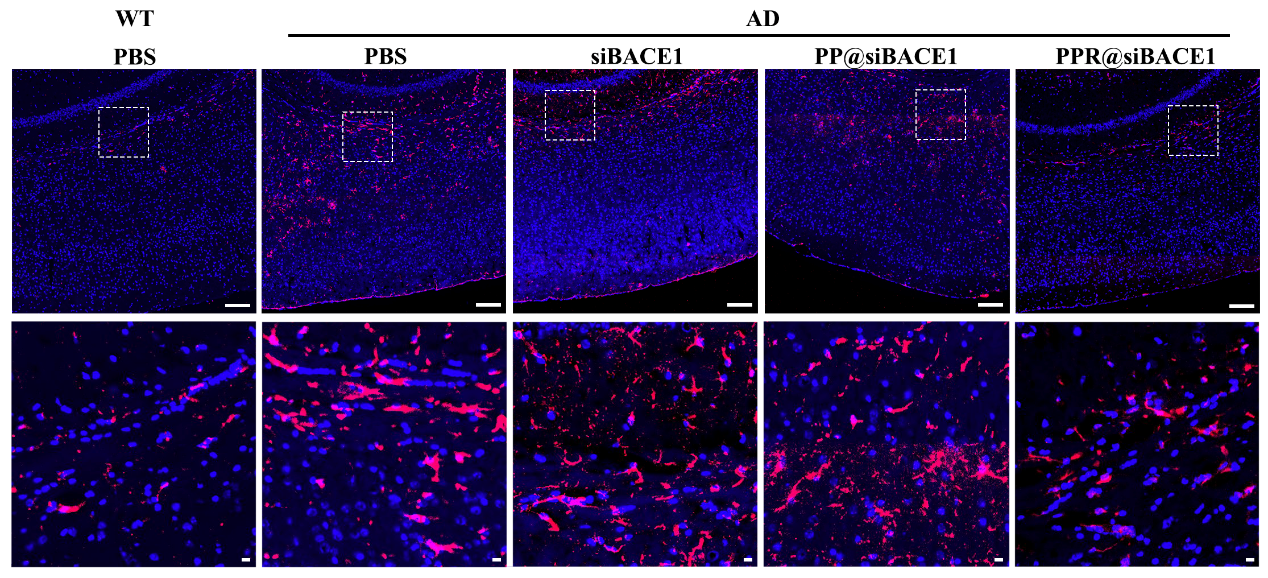


**Fig. S17.** Immunofluorescence staining of astrocyte with GFAP (red) in the cortex region of mice from different groups. Scale bars: 100 μm (original figure), 5 μm (magnified inset).


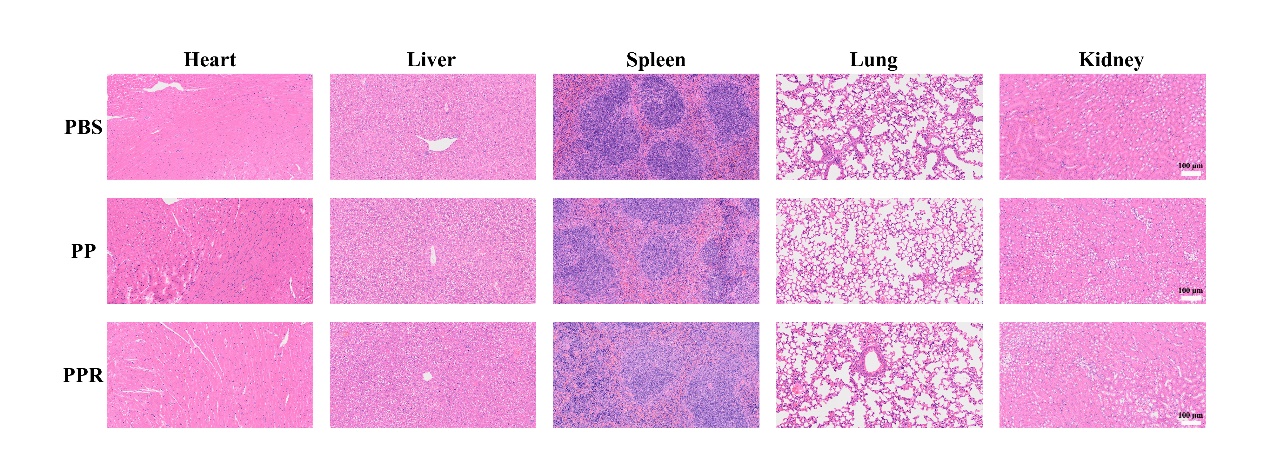


**Fig. S18.** Representative data for hematoxylin and eosin staining in major organs of mice from different groups. Scale bar, 100 μm.

**Table S1.** qPCR primer sequences

| ***gene*** | **Forward (5’-3)** | **Reverse (5’-3)** |
| --- | --- | --- |
| *Bace1* | TACTACTGCCCGTGTCCACC | ACAACCTGAGGGGAAAGTCC |
| *Gapdh* | TTGATGGCAACAATCTCCA | CGTCCCGTAGACAAAATGGT |
| *Il-1β* | GAAATGCCACCTTTTGACAGTG | TGGATGCTCTCATCAGGACAG |
| *Il-6* | TAGTCCTTCCTACCCCAATTTCC | TTGGTCCTTAGCCACTCCTTC |
| *Tnf-α* | TCTTCTCATTCCTGCTTGTGG | TCTTCTCATTCCTGCTTGTGG |
