# Supplementary figures and images for: Targeted delivery of BACE1 siRNA for synergistic treatment of Alzheimer's disease
Source: Transl Neurodegener. 2025 Aug 14;14:41. doi: 10.1186/s40035-025-00503-7 (PMC12351871; doi:10.1186/s40035-025-00503-7)

**Figure3.f:**


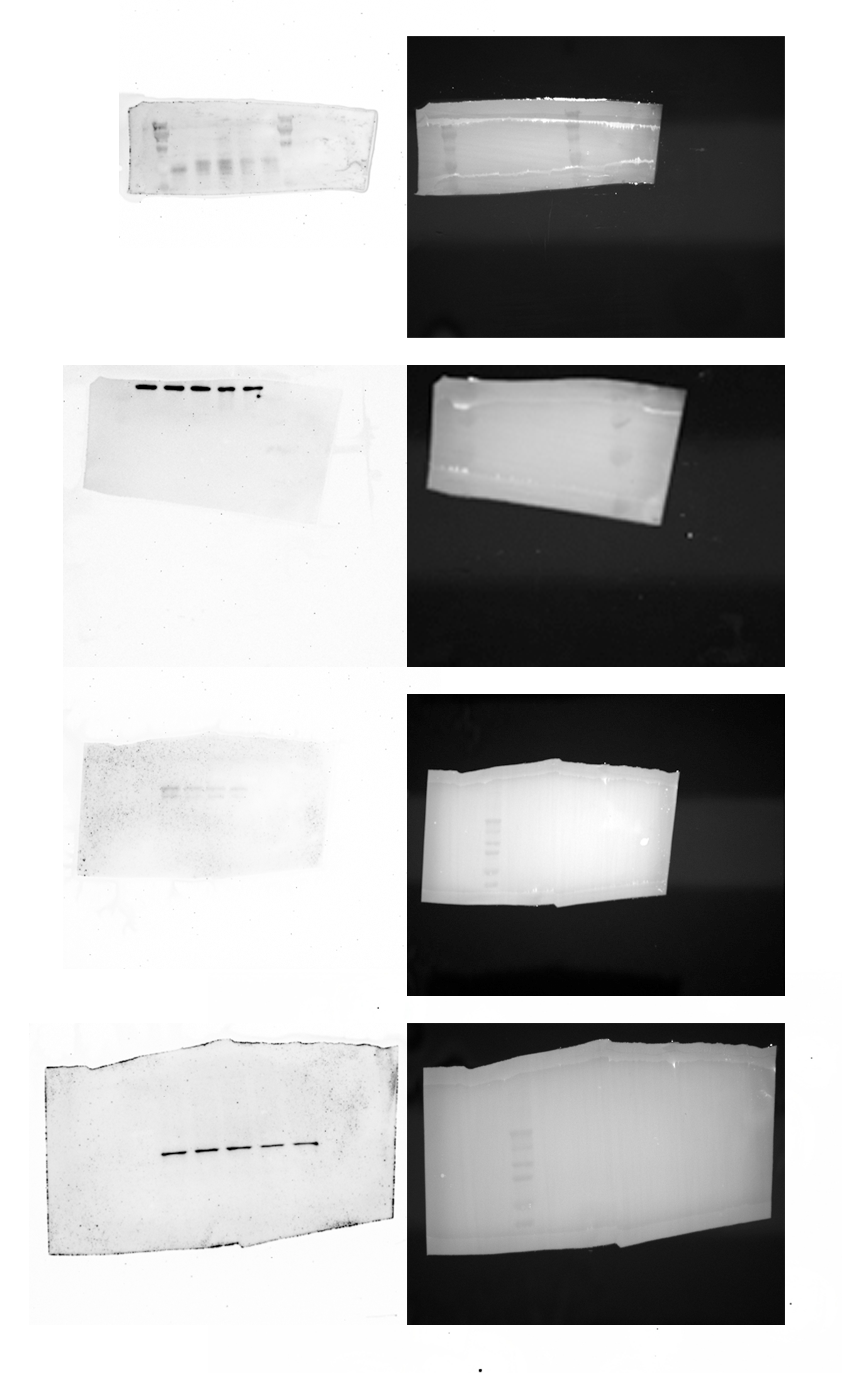


**FigureS.7:**


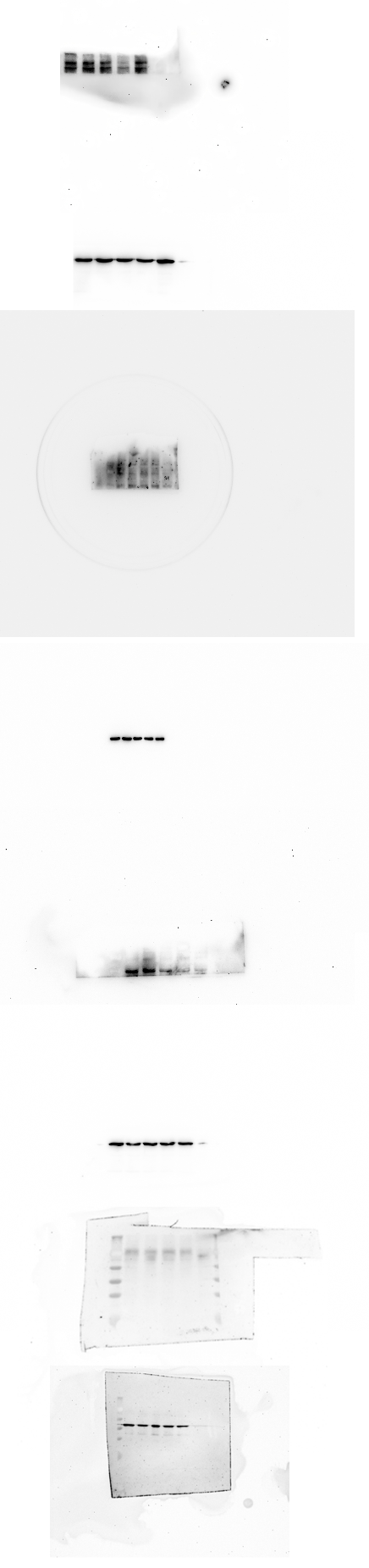


**Figure6.b:**

**
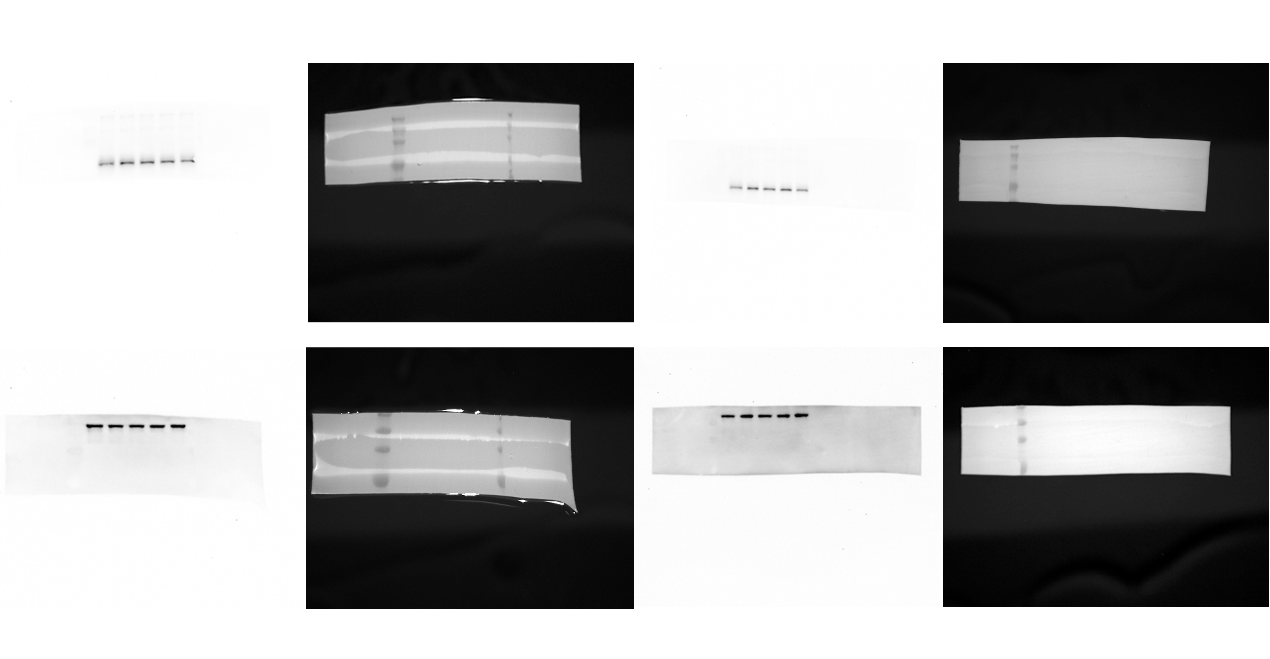
**

**Figure6.c:**


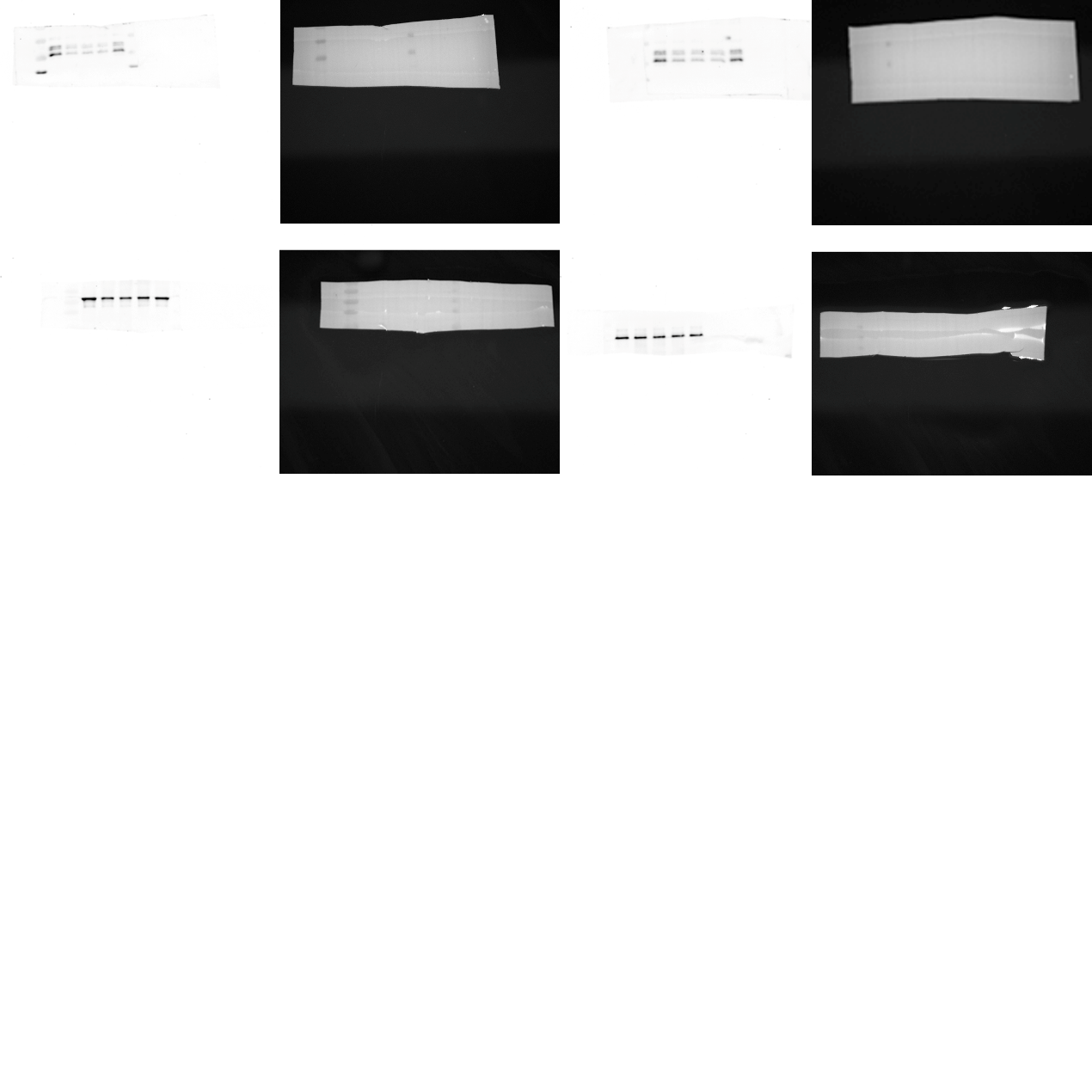


**FigureS.18a:**

**
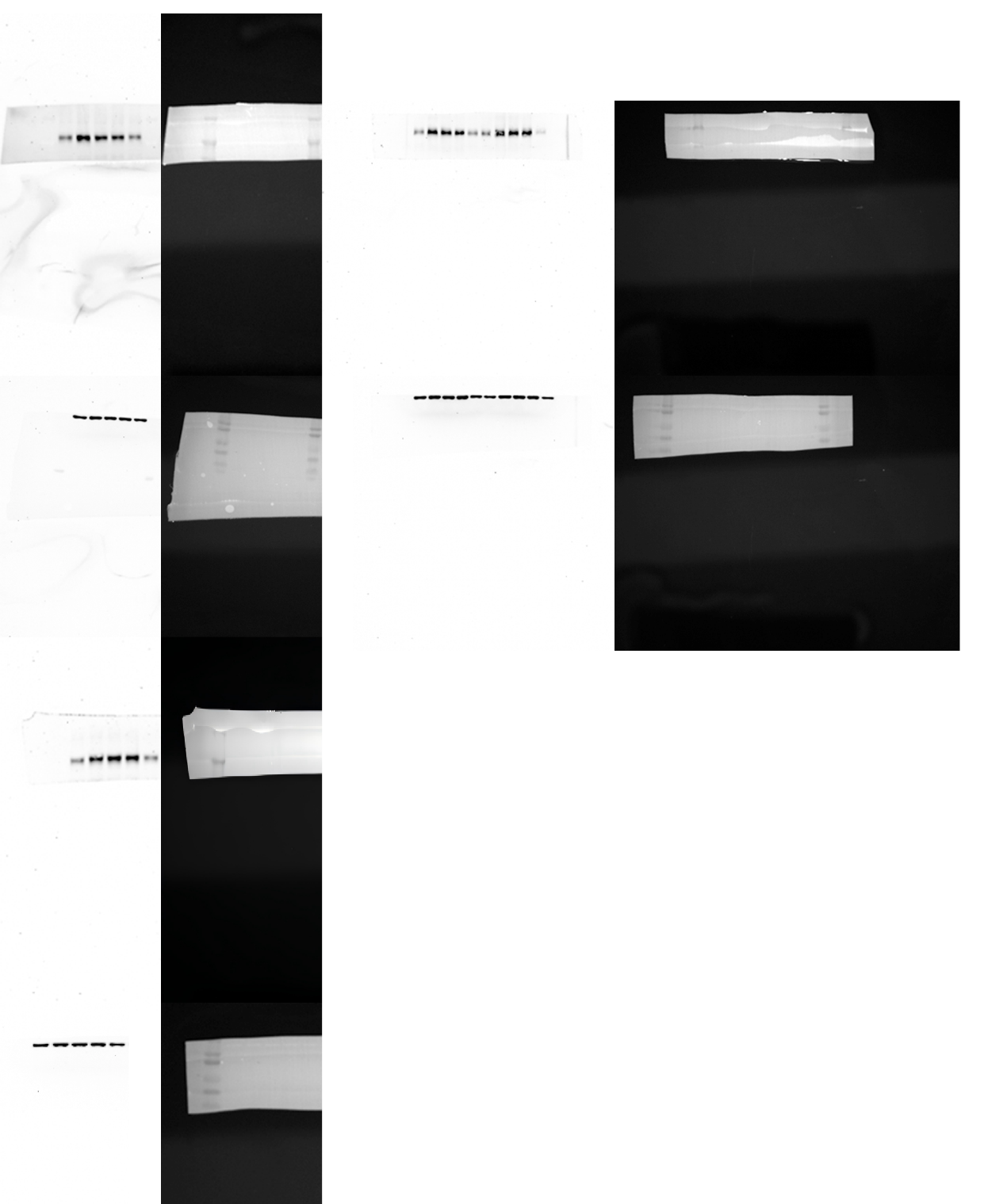
**

**FigureS.18b:**


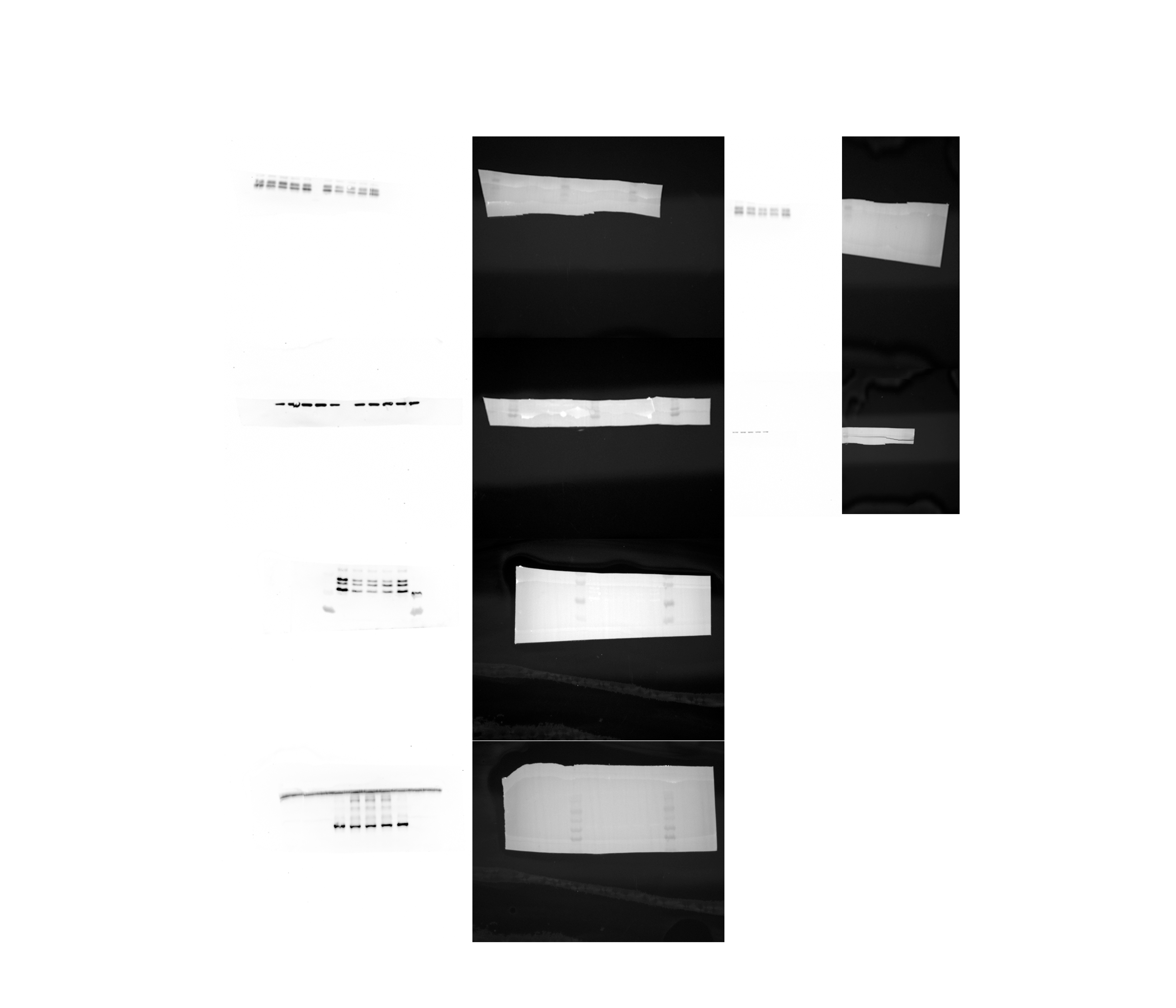

Supplement: Supplementary file 2 — Additional file 2 (DOCX 7219 KB) Uncropped immunoblot images. [file 40035_2025_503_MOESM2_ESM.docx]
